# Prebiotic Xylo-Oligosaccharides Modulate the Gut Microbiome to Improve Innate Immunity and Gut Barrier Function and Enhance Performance in Piglets Experiencing Post-Weaning Diarrhoea

**DOI:** 10.3390/microorganisms13081760

**Published:** 2025-07-28

**Authors:** James S. Stanley, Stephen C. Mansbridge, Michael R. Bedford, Ian F. Connerton, Kenneth H. Mellits

**Affiliations:** 1 Division of Microbiology, Brewing, and Biotechnology, School of Biosciences, University of Nottingham, Sutton Bonington LE12 5RD, UK; ian.connerton@nottingham.ac.uk; 2 The National Institute of Poultry Husbandry, Harper Adams University, Newport TF10 8NB, UK; smansbridge@harper-adams.ac.uk; 3 AB Vista, Marlborough SN8 4AN, UK; mike.bedford@abvista.com

**Keywords:** prebiotics, oligosaccharides, dietary carbohydrates, pigs, growth, microbiota, intestinal health, histology, immunity

## Abstract

During commercial pig production, weaning is a major stressor that disrupts the gut microbiome, compromises intestinal barrier integrity, and increases the susceptibility of piglets to pathogens. This often results in post-weaning diarrhoea (PWD), leading to growth retardation, morbidity, and economic loss. This study investigated the effects of dietary xylo-oligosaccharide (XOS) supplementation on the growth performance and gut health of 216 piglets with naturally occurring PWD. Piglets received either 0 (CON), 50 (XOS-50), or 500 (XOS-500) mg XOS/kg feed from weaning at 28 days of age (d1) for 54 days. XOS-500 significantly improved body weight at d22 and d54, but had no effect on average daily gain, daily feed intake (DFI), or feed conversion ratio. The intestinal microbiota alpha-diversity was unaffected by XOS, though jejunal beta diversity differed between CON and XOS-500 groups at d22. Jejunal Chao richness correlated positively with d54 body weight, while ileal Chao richness correlated negatively with DFI. *Salmonella* was present in all diet groups but did not differ in abundance; however, the levels were negatively correlated with alpha diversity. XOSs increased *Lactobacillus* (d22, d54) and *Clostridium_XI* (d22), while reducing *Veillonellaceae* spp. (d22). XOSs reduced jejunal goblet cell (GC) density at d22 but increased duodenal and jejunal GCs and reduced duodenal crypt depth at d54. XOSs upregulated the genes for the tight junction proteins CLDN2, CLDN3, ALPI, and ZO-1, while downregulating the cytokine IL-8. These findings highlight XOSs’ potential to improve growth and gut health in weaning piglets with naturally occurring PWD, to maintain productivity and enhance welfare.

## 1. Introduction

Global meat protein consumption is expected to increase by 14% by 2030 compared to the 2018–2020 baseline average, with pigmeat accounting for 33% of this growth and total consumption projected to reach 127 million metric tonnes [1]. Given the rising demand, optimising animal performance and feed conversion efficiency is essential for ensuring both economic sustainability and productivity in commercial pig production.

During suckling, sow colostrum provides piglets with maternal antibodies that can be absorbed through their immature gut, conferring passive immunity before gut closure occurs within the first 48 h of life [2]. Moreover, oligosaccharides found in sow’s milk are utilised by the neonatal piglet’s resident microbiota to promote a healthy gastrointestinal tract (GIT) microbiome, prevent pathogen colonisation of the gut, and modulate immune responses [3]. Naturally, piglets begin weaning at 10 to 12 weeks of age, coinciding with intestinal maturation—far later than in commercial pig production systems, where weaning occurs at just 3 to 4 weeks, before full GIT development [4]. This early weaning disrupts normal gut maturation and exposes piglets to multiple stressors, including abrupt separation from the sow, social mixing with unfamiliar littermates, establishment of a new hierarchy, and a sudden transition from palatable, highly digestible sow milk to a less digestible, cereal-based commercial diet [5]. Consequently, piglets display a transient drop in voluntary feed intake, leading to a post-weaning growth check (PWGC), villus atrophy, reduced digestive enzyme activity, gut microbiota dysbiosis, and gut inflammation [5,6,7]. This disruption of the gut can compromise colonisation resistance, predisposing piglets to enteric pathogens, such as enterotoxigenic and enteropathogenic *Escherichia coli* as well as *Salmonella* spp. [8]. These pathogens are major contributors to post weaning diarrhoea (PWD), a leading cause of morbidity and mortality in piglets, resulting in significant economic losses for producers [6].

Sub-therapeutic doses of antibiotics were routinely employed in intensive pig production systems to combat PWD and the PWGC [9,10]. However, the inclusion of antibiotics as growth promoters (AGPs) was banned in the European Union (EU) under Regulation (EC) No 1831/2003, effective from 2006, due to growing concerns over antimicrobial resistance and the potential transfer of antibiotic resistance genes among bacteria, which may subsequently be transmitted from animals to humans [11]. Various feed additives—such as prebiotics, probiotics, essential oils, organic acids, herbs, and exogenous enzymes—have been proposed and are currently being investigated as alternatives to AGPs [12]. The focus has now shifted towards optimising their use in practice to enhance gut health and mitigate weaning-associated issues, such as inflammation and diarrhoea.

Prebiotics are defined as “a substrate that is selectively utilised by host microorganisms conferring a health benefit” [13]. Xylo-oligosaccharides (XOSs) are non-digestible carbohydrates that have been shown to stimulate beneficial bacteria, such as *Bifidobacterium* and *Lactobacillus*, in vitro and throughout the GIT of the weaning pig [14,15,16,17,18]. XOSs are synthesised through the enzymatic or chemical hydrolysis of xylan, a major component of plant cell walls, generating oligosaccharides of varying chain lengths, typically composed of two to six xylose monomers linked via β-1,4-glycosidic bonds [19]. Several studies have reported that XOS supplementation can enhance performance, reduce intestinal inflammation, and modulate the gut microbiota in healthy weaning piglets, by promoting beneficial bacteria while suppressing potentially pathogenic species [18,20,21,22]. Additionally, XOS has been shown to alleviate *E. coli* lipopolysaccharide-induced intestinal injury in weaned piglets by improving gut morphology, suppressing inflammatory cytokines, and modulating the GIT microbiota [23,24]. Moreover, XOS has been shown to alleviate *Salmonella*-induced inflammation by increasing *Bifidobacterium animalis* and inhibiting *Salmonella* colonisation in a murine challenge model [25]. These findings indicate that XOS contributes to improved gut microbiota composition and may offer protection against enteric pathogens that contribute to morbidity and diarrhoea in weaning piglets.

Lee et al., (2025) [26] reviewed the use of XOS as a sustainable alternative to AGPs in both healthy and pathogen-challenged pigs across multiple production stages, including the pre-weaning, post-weaning, nursery, and growing/fattening phases. The authors demonstrated that XOS functions as a promising feed additive capable of enhancing growth performance and gut health by modulating the gut microbiota, improving intestinal architecture, attenuating inflammatory responses, and reinforcing gut barrier integrity. While the effects of XOS in healthy weaning pigs have been well-documented, fewer studies have examined its impact in pathogen-challenged piglets, and, notably, no studies to date have assessed the effects of XOS in weaning piglets experiencing PWD under commercial farm conditions that reflect real-world production settings. We hypothesise that XOS supplementation will alleviate the PWGC observed during weaning, which is exacerbated by environmental PWD, through the modulation of the GIT microbiome and the improvement of gut barrier function and morphology. Therefore, the objectives of this study were to investigate the effects of XOS supplementation on growth performance, gut microbiome composition, immune response, gut barrier integrity, intestinal architecture, and goblet cell (GC) expression in weaning piglets exhibiting self-limiting PWD that did not require antibiotic or other therapeutic intervention. The concentrations of in-feed XOS used in this study were selected based on previous reports demonstrating beneficial effects of XOS supplementation on the performance and gut health of weaning pigs. A higher inclusion level of 500 mg XOS per kg of feed reflects doses commonly employed in such studies [16,17,18], while a lower level of 50 mg XOS per kg was included to evaluate potential effects at a sub-therapeutic dose that may still positively influence host health under commercial production conditions.

## 2. Materials and Methods

### 2.1. Ethical Approval

All experimental procedures were approved by the Harper Adams University Research Ethics Committee (Approval Reference Number: 1388-202112-STAFF) and the University of Nottingham Animal Welfare and Ethical Review Body (Approval Reference Number: 40). Animals were humanely killed in accordance with the requirements of the Animals (Scientific Procedures) Act 1986 (as amended).

### 2.2. Experimental Animals and Trial Design

A total of 216 piglets (JSR 9T dam × JSR Tempo sire) with an average weaning weight of 8.31 ± 0.05 kg were used to investigate the effects of in-feed XOS on growth performance and gut health compared to a basal diet with no XOS supplementation. On day 1 of weaning (d1), piglets were randomly assigned to one of the following three dietary groups: (1) a control basal diet (CON), (2) the CON diet supplemented with 50 mg of XOS per kg of feed (XOS-50), or (3) the CON diet supplemented with 500 mg of XOS per kg of feed (XOS-500). Piglet allocation to diets and diet assignment to pens were performed using a computer-based random number generator. Each diet group had 12 replicate pens, with 6 pigs per pen (balanced across sex). Sample size was determined a priori using the procedure of Berndtson (1991) [27], aiming to detect a 10% difference in body weight with a coefficient of variation of 5–8%, based on previous studies. The calculation was performed with 80% power (β = 0.2) and a significance level of 0.05 (α), in accordance with EFSA FEEDAP (2018) guidelines [28]. The trial lasted for 54 days. Feed and water were provided ad libitum. Environmental enrichment was provided via chew toys and chains, while compressed wood blocks or edible materials were avoided to prevent potential microbiome interference. The study was conducted at the Harper Adams University Future Farm Pig Unit. Neonatal piglets were housed with their sows in farrowing pens (4.3 m^2^) heated with industry-standard heat lamps. Upon weaning, pigs were transferred to group housing in pens (2.45 m^2^) with plastic slatted floors and industry-standard heating. The nursery temperature was set at 28 °C on d1 and gradually decreased following a controlled temperature curve, reaching 20 °C by d54. A light/dark cycle was maintained from 08:00 h to 16:00 h. All piglets displayed clinical signs of diarrhoea within the first week of weaning, indicative of a PWD outbreak. However, the infection was deemed self-limiting by a veterinary surgeon, and no antibiotic intervention was administered. On d1, pigs were vaccinated with Porcilis PCV M Hyo (Intervet International BV, MSD Animal Health, Boxmeer, The Netherlands; Vm:EU/2/08/091/001-010) for active immunisation against Porcine circovirus type 2 and *Mycoplasma hyopneumoniae*. Xylo-oligosaccharides with degree of polymerisation >2 were obtained from corn cob with a purity of 37.3% and supplied by AB Vista (Marlborough, UK). The feeding regime was as follows: starter phase (d1–d7), linker phase (d8–d22), and grower phase (d23–d54). All diets were formulated to meet or exceed nutritional requirements and were free from growth promoters, antibiotics, or probiotics. Diet formulations for the pre-weaning creep diet and post-weaning study diets are provided in Tables S1 and S2. At birth, piglets were ear-tagged for identification and housed in litters with continuous access to a sow for the first 28 days of age (da). From birth to weaning (28 da), piglets were offered supplementary milk (Faramate, Volac International Ltd., Royston, UK) and creep feed from 15 da until weaning. Sows were vaccinated with Porcilis^®^ Porcoli Diluvac Forte (Intervet International BV; Vm:EU/2/96/001/003-008) three weeks before farrowing to provide passive immunity against neonatal enterotoxicosis. Animals were not subject to procedures that may cause pain, suffering, distress, or lasting harm exceeding the lower threshold, as defined under the Animals (Scientific Procedures) Act 1986 (as amended). Pigs were monitored daily throughout the trial for signs of ill health, including diarrhoea, lameness, respiratory distress, and failure to gain weight. Humane endpoints included sustained weight loss, severe lameness, or systemic illness, at which point animals were removed from the study and treated or euthanised in consultation with the named animal welfare officer or named veterinary surgeon. Expected mortality from non-procedure-related causes was ≤5%, with deaths recorded alongside weight, date, and suspected cause. No adverse effects of XOS supplementation were observed throughout the study, with pigs in both XOS-50 and XOS-500 groups maintaining normal feed intake and growth patterns comparable to other trials conducted at the Harper Adams University Future Farm Pig Unit. Pigs were weighed on days 1, 7, 14, 22, and 54 to monitor production performance. On sampling days (d22 and d54), pigs were weighed immediately before being humanely killed. One pig per pen (selected as the closest to the average pen weight) was euthanised using a penetrative captive bolt and pithing, after which GIT luminal digesta and tissue were collected for laboratory analysis. Investigators were not blinded during the farm trials to ensure correct diet administration to each pen; however, they were blinded to dietary group during laboratory and statistical analyses. Feed intake was recorded throughout the study. Average daily gain (ADG), daily feed intake (DFI), and feed conversion ratio (FCR) were calculated on a pen basis as follows: ADG = total weight gain/total study days; DFI = total feed intake/total study days; and FCR = total feed intake/total weight gain (corrected for mortality).

### 2.3. Sample Collection

Upon death, digesta were aseptically collected from the piglet duodenal, jejunal, ileal, caecal, colonic, and rectal lumens. Samples were immediately stored on dry ice before transfer to the laboratory and held at −80 °C for long term storage until bacterial DNA isolation. Cross-sections of the duodenal, jejunal and ileal tissue were excised post-mortem and immediately preserved in pre-filled 10% neutral buffered formalin (NBF) specimen containers (Fisher Scientific, Loughborough, UK) for histological analysis and GC enumeration. Intestinal tissue was excised post-mortem from the midpoints of the duodenum, jejunum, and ileum, as well as from the apex of the caecum and colon, for mRNA expression analysis. These tissue biopsies were taken into cryotubes containing 1.5 mL RNAlater RNA Stabilisation Reagent (Invitrogen, Thermo Fisher Scientific, Loughborough, UK), snap frozen in liquid nitrogen, transported to the laboratory, and held at −80 °C until RNA isolation.

### 2.4. DNA Isolation and PCR Amplification of 16S rRNA Gene Sequences

Bacterial DNA was isolated from 200 mg of luminal contents using the QIAamp 96 PowerFecal QIAcube HT Kit and the QIAcube HT (Qiagen, Hilden, Germany) according to the manufacturer’s instructions, with the following modifications. The weighed digesta was homogenised using Pathogen Lysis Tubes S (Qiagen, Hilden, Germany) on the FastPrep-24 5G bead-beater system (MP Biomedicals, Solon, OH, USA). For each sample, the V4 region of the bacterial 16S rRNA genes were polymerase chain reaction (PCR) amplified using the primers 515f (5′ GTGCCAGCMGCCGCGGTAA 3′) and 806r (5′ GGACTACHVGGGTWTCTAAT 3′) [29]. Amplicons were sequenced on the Illumina MiSeq platform using 2 × 250 bp cycles (Illumina, Cambridge, UK). Sequence data were deposited in the NCBI database within the BioProject PRJNA1240184, with SRA records available at https://www.ncbi.nlm.nih.gov/sra/PRJNA1240184 (accessed on 22 March 2025).

### 2.5. Microbiota Diversity Analysis

The 16s rRNA gene sequences were quality filtered and clustered into operational taxonomic units (OTUs) using Mothur v.1.48.1, following the MiSeq SOP (https://www.mothur.org/wiki/MiSeq_SOP, accessed on 4 November 2024) [30,31]. Sequences were aligned against a reference alignment from the SILVA rRNA database (version 102; available at https://mothur.org/wiki/silva_reference_files accessed on 5 March 2025) [32]. OTUs were clustered using the “OptiClust” algorithm with a 0.03 similarity cut-off [33]. The consensus taxonomy for each OTU was assigned using the “classify.otu” command in Mothur, with reference data from the Mothur-formatted version 9 of the Ribosomal Database Project [34,35]. Rarefaction curves were generated using resampling without replacement to assess sampling effort. Syntaxes for use in Mothur are deposited at https://github.com/J-S-Stanley/XOS-2025 (accessed on 25 March 2025).

### 2.6. Salmonella Enrichment

Pre-enrichment of *Salmonella* was performed in triplicate for each sample, with 1 g of digesta inoculated into a conical flask containing 99 mL of buffered peptone water (BPW) and incubated at 37 °C for 18 h. In total, 1 L of BPW contained 10 g of peptone, 5 g of sodium chloride, 3.5 g of disodium phosphate, and 1.5 g of potassium dihydrogen phosphate. For each pre-enrichment culture, 3 × 300 µL aliquots from the same BPW flask were spotted onto a Modified Semi-Solid Rappaport Vassiliadis (MSRV) plate and incubated at 42 °C for 24 h for selective enrichment of *Salmonella*. MSRV plates with no visible signs of *Salmonella* growth at 24 h were incubated for an additional 24 h (48 h total) to confirm the absence of motile colonies. In total, 1 L of MSRV media contained 4.6 g tryptose, 4.6 g casein hydrolysate, 7.34 g sodium chloride, 1.47 g potassium dihydrogen phosphate, 10.93 magnesium chloride (anhydrous), 0.037 g malachite green oxalate, and 2.7 g agar.

### 2.7. Quantification of Lactobacillus HSP60 and Salmonella invA Genes

Bacterial DNA was isolated from pig digesta samples as previously described. Modified primers targeting the heat-shock protein 60 gene (*HSP60*) were used to enumerate *Lactobacillus* abundance, and primers targeting the invasion protein A gene (*invA*) were used to enumerate *Salmonella* abundance in each sample. The expression of target genes was determined using quantitative PCR (qPCR) with the Luna^®^ Universal qPCR Master Mix kit (New England Biolabs, Ipswich, MA, USA) on the QIAquant 96 PCR thermal cycler (Qiagen, Hilden, Germany), following manufacturer’s instructions. The primer sequences for *HSP60* and *invA* are presented in Table S3.

### 2.8. RNA Isolation, RT-qPCR, and Gene Expression Analysis

Pig intestinal RNA was isolated from duodenal, jejunal, ileal, caecal, and colonic tissue. For each sample, 15 mg tissue was homogenised in 600 µL Buffer RLT (Qiagen, Hilden, Germany) containing 1% (*v*/*v*) β-mercaptoethanol using 2 mL nuclease-free Lysing Matrix D tubes (MP Biomedicals, Solon, OH, USA) on the FastPrep-24 5G bead-beater system (MP Biomedicals, Solon, OH, USA). Total RNA was then purified using the QiAamp RNeasy 96 QIAcube HT Kit and the QIAcube HT (Qiagen, Hilden, Germany) according to the manufacturer’s instructions. Purified RNA was eluted in nuclease-free water, validated for quantity and quality using UV spectrophotometry (Biochrom BioDrop Duo+; Biochrom, Harvard Bioscience, Inc., Holliston, MA, USA), and stored at −80 °C until reverse transcription qPCR (RT-qPCR) within 1 week. RNA was reverse transcribed using the RT^2^ First Strand Kit to synthesise cDNA and remove residual genomic DNA according to manufacturer’s instructions (Qiagen, Hilden, Germany). Primer sets were purchased through the Custom RT^2^ Profiler PCR Assays service (Qiagen, Hilden, Germany), with primers pre-attached to the plate for the detection of the porcine genes encoding gut barrier proteins *OCLN* (occludin), *TJP1* (zonula occludens-1; ZO-1), *TJP2* (zonula occludens-2; ZO-2), *CLDN2* (claudin-2), and *CLDN-3* (claudin-3); the cytokines *IL-1*β (interleukin-1β), *IL-6* (interleukin-6), and *IL-10* (interleukin-10); the chemokine *IL-8* (interleukin-8); and the brush-border enzyme *ALPI* (intestinal alkaline phosphatase). The housekeeping genes (HKG) *GAPDH* (glyceraldehyde-3-phosphate dehydrogenase) and *RPL4* (ribosomal protein L4) were also quantified. The expression of host target genes was determined using qPCR with the RT^2^ SYBR Green qPCR Mastermix kit (Qiagen, Hilden, Germany) using the Roche Diagnostics LightCycler 480 Instrument (Hoffman La Roche, Basel, Switzerland). For each sample, the ct values of both HKGs were used to calculate an average HKG ct value. Target gene transcript levels and fold changes were determined using the 2^−ΔΔct^ method. Here, ct represents the qPCR cycle threshold, Δct is the difference between the target gene ct value and the average HKG ct value, and ΔΔct is the difference in transcript levels between pigs on the control and XOS diets. Statistical analysis was performed on the final 2^−ΔΔct^ values.

### 2.9. Histology Analysis

Intestinal cross-sections fixed in 10% NBF were processed using a Epredia™ Excelsior™ AS tissue processor (Epredia Holdings Ltd., Portsmouth, NH, USA). Samples underwent a series of alcohol washes to dehydrate the tissue. Samples were then cleared using xylene to remove residual ethanol and facilitate paraffin infiltration. Tissues were subsequently embedded in molten paraffin wax, allowed to solidify, and sectioned at 5 µm thickness using a microtome. Sections were mounted on glass slides and stained using the periodic acid–Schiff (PAS) method. Following staining, all PAS-stained slides were scanned using the NanoZoomer Digital Pathology (NDP) system at 40× magnification (Hamamatsu, Welwyn Garden City, UK). Histological analysis was performed using NDP.view2 software (version 2.9.29) (Hamamatsu, Welwyn Garden City, UK), where all well-oriented villi and crypts in each tissue section were identified. From these, up to ten villi and crypts were randomly selected for measurement using a random number generator. The NDP.view2 software functions were used to measure villus height, crypt depth, villus area, and crypt area, as well as to enumerate GC’s in both villi and crypts. Villus height was measured from the villus tip to the crypt opening, while crypt depth was measured from the crypt opening to the base of the crypt. The villus-to-crypt ratio (VCR) was calculated by dividing villus length by crypt depth.

### 2.10. Statistical Analyses

All statistical analyses and figure generation were performed using R version 4.2.1 [36] in RStudio version 2023.06.0+421 [37] unless otherwise stated. Normality was assessed using the Shapiro–Wilk test [38], and parametric or non-parametric methods were applied accordingly. Multiple comparisons (Tukey’s honest significant difference (HSD) and Dunn’s tests) were adjusted for false discovery rates using the Benjamini–Hochberg procedure [39]. A repeated measures analysis of variance (RM-ANOVA) was used to assess differences in pig body weight (BW) over time, while ANOVA was used to compare BW on d1, d7, d14, d22, and d54, as well as ADG, DFI, and FCR between diets, with Tukey’s HSD test for pairwise comparisons. For all microbiome analyses, only samples containing a total number of sequences greater than or equal to the indicated subsampling depth were included, to ensure sufficient sequencing depth and data quality. Good’s coverage [40] and α-diversity indices (Chao richness [41] and inverse Simpson diversity [42]) were calculated using the “summary.single” command in Mothur. Differences in α-diversity were tested using Kruskal–Wallis tests with Dunn’s test for post hoc comparisons. β-diversity indices (Yue and Clayton dissimilarity [43], Bray–Curtis dissimilarity [44], and Jaccard similarity [45]) were calculated and assessed for significance using analysis of molecular variance (AMOVA) in Mothur [46,47]. Spearman’s rank correlation was used to assess relationships between alpha diversity indices and performance metrics (d54 BW, ADG, DFI, and FCR) with linear regression analysis performed using linear modelling. OTU-level abundances were obtained from Mothur output files, while relative abundances were calculated in R. Differential abundance analysis of bacterial taxa was performed using ALDEx2 (version 1.30.0) and ANCOM-BC2 (version 2.0.3) in R, applying a minimum prevalence threshold of 10% and a minimum sequencing depth of 50 [48,49]. ALDEx2 was run using the “aldex.clr” approach. Syntaxes for ALDEx2 and ANCOM-BC2 analyses in R are deposited at: https://github.com/J-S-Stanley/XOS-2025 (accessed on 25 March 2025). Kruskal–Walis tests were used to compare from the 16s rRNA sequencing data total *Lactobacillus* abundance between diet groups for each timepoint and GIT site. For *HSP60* and *invA* gene expression, samples with ct values > 35 were excluded from analysis, where ct represents the PCR cycle threshold. *HSP60*/*invA* ratios were calculated by dividing the *HSP60* ct value by the *invA* ct value for each sample. These ratios were then correlated with alpha diversity indices using Spearman’s rank correlation. Differences in villus height, crypt depth, VCR, and GC density between diets were analysed using Kruskal–Walis tests with Dunn’s test for post hoc comparisons. For gene expression analysis, ct values > 35 were excluded from analysis, and outliers were identified and removed if they fell below Q1 − (1.5 × IQR) or above Q3 + (1.5 × IQR) before analysis using Kruskal–Wallis and Dunn’s tests.

## 3. Results

### 3.1. Production Performance

**Figure 1:**
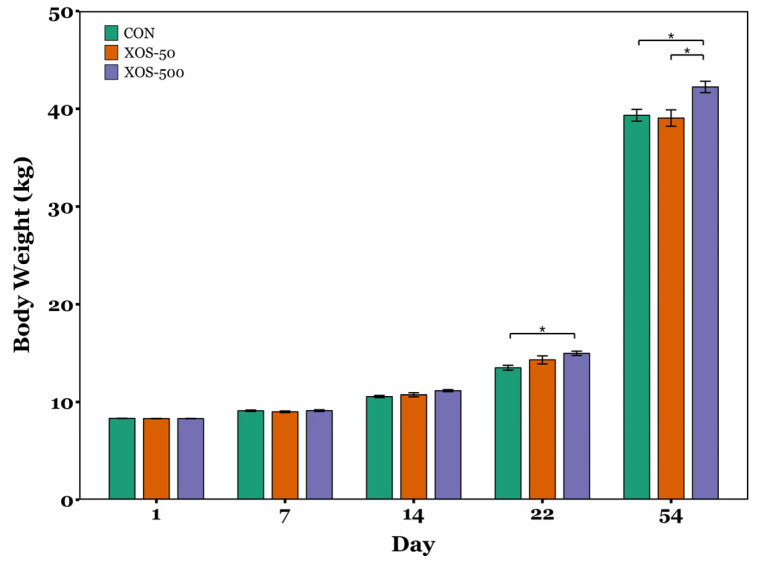
Body weights of control and XOS-fed piglets at d1, d7, d14, d22, and d54 post-weaning. Bars represent the average body weight across the replicate pens for each diet. Error bars represent standard error of the mean. *n* = 12 pens per diet group. Asterisks indicate statistically significant pairwise differences between diet groups as determined by Tukey’s HSD test: *, *p* < 0.05.

**Figure 2:**
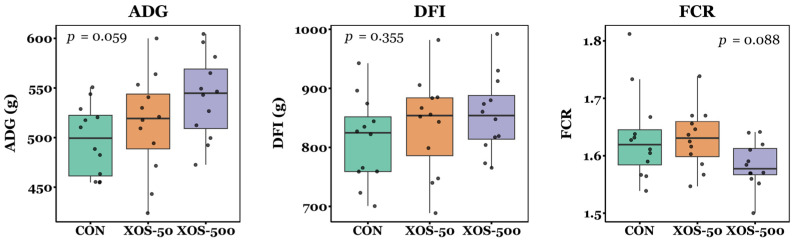
ADG, DFI, and FCR of piglets on control or XOS diets from d1 to d54. Boxplots display the median, first quartile, and third quartile of ADG, DFI, and FCR. Box whiskers extend to the smallest and largest values within 1.5 times the interquartile range. *n* = 12 pens per diet group. *p*-values from Kruskal–Wallis tests.

Animals were reared in a commercial production facility. Pig BW on days 1, 7, 14, 22, and 54, and ADG, DFI, and FCR were normally distributed according to the Shapiro–Wilk tests (*p* > 0.05). The RM-ANOVA indicated that there was a significant effect of both diet and time on pig BW across the study (*p* < 0.05), indicating that BW changed over time and was influenced by diet group. There was no significant difference in BW between diet groups at d1 and d7 (*p* > 0.05, ANOVA; Figure 1). By d14, ANOVA detected a significant difference in BW (*p* = 0.02); however, pairwise comparisons showed no significant differences between diet groups (*p* > 0.05, Tukey’s HSD test). ANOVA also detected significant differences between diet groups on d22 and d54 (*p* < 0.05). On d22, BW was significantly greater in pigs receiving the XOS-500 diet compared to controls (*p* = 0.014). By d54, BW was significantly greater in XOS-500 pigs compared to both controls and XOS-50 animals (*p* = 0.022 and *p* = 0.021, respectively). The ADG, DFI, and FCR did not differ significantly between diet groups during the study (*p* > 0.05, ANOVA; Figure 2).

### 3.2. GIT Microbiota Diversity

A total of 8,087,038 high-quality V4 16s rRNA sequence reads were obtained from 432 piglet GIT samples. Samples from the upper and lower GIT were subsampled to 5488 and 6829 sequences per sample, respectively, to achieve a Good’s coverage of 97.6–99.9% (minimum to maximum across all samples). Rarefaction curves generated using resampling without replacement reached or approached asymptotes for all samples, indicating sufficient sampling depth was achieved (Figure S1).

Inverse Simpson diversity and Chao richness were normally distributed for most samples (*p* > 0.05; Shapiro–Wilk tests), but not all. No significant differences between inverse Simpson diversity or Chao richness were observed throughout the study (Kruskal–Wallis tests; Table 1; supplementary boxplots provided in Figure S2).

Significant differences in the β-diversity of jejunal communities were observed between CON and XOS-500 pigs on day 22 for Yu and Clayton (*p* = 0.005) and Bray–Curtis (*p* = 0.004) distances, but not for Jaccard similarity, as determined by AMOVA (Table 2 and Figure S3). No other significant differences in β-diversity were observed during the trial (*p* > 0.05).

### 3.3. Performance and Microbiota Diversity Correlation

**Figure 3:**
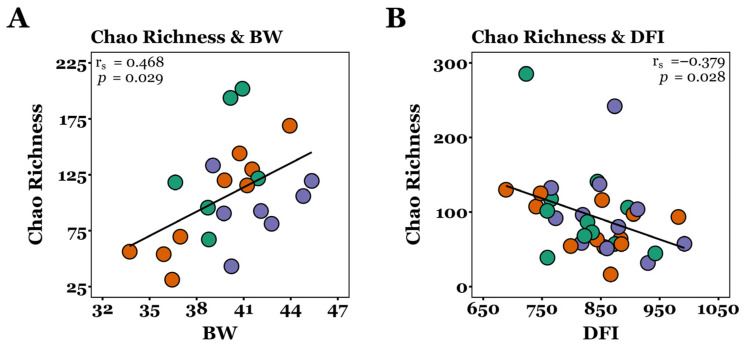
Correlations between d54 alpha diversity and performance metrics. (**A**) Jejunal Chao richness and d54 body weight for CON (*n* = 6), XOS-50 (*n* = 9), and XOS-500 (*n* = 7) pigs. (**B**) Ileal Chao richness and DFI from d1 to d54 for CON (*n* = 11), XOS-50 (*n* = 12), and XOS-500 (*n* = 11) pigs. Green points, CON; orange points; XOS-50; purple points, XOS-500. r_s_, Spearman’s rank correlation coefficient; *p*, Spearman’s rank test *p*-value.

Final BW (d54) and jejunal Chao Richness were positively correlated (r_s_ = 0.468, *p* = 0.029, Spearman’s rank correlation; Figure 3), with linear modelling showing a significant relationship (R^2^ = 0.220, *p* = 0.028). Additionally, DFI decreased as ileal Chao Richness increased (r_s_ = −0.379, *p* = 0.028, Spearman’s rank), with linear modelling revealing a significant relationship (R^2^ = 0.134, *p* = 0.034). There was no significant correlation between ADG or FCR and Chao richness, nor between final BW, ADG, DFI, or FCR and inverse Simpson diversity (*p* > 0.05; Spearman’s rank).

### 3.4. GIT Microbiota Composition

**Figure 4:**
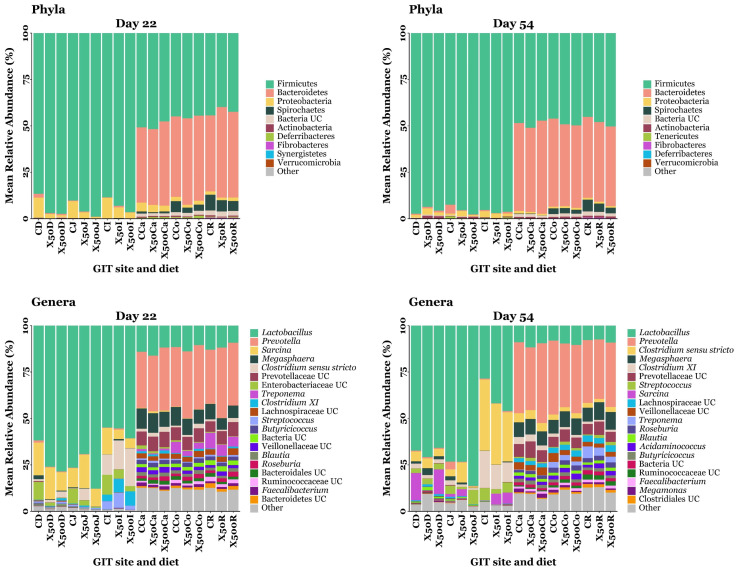
The relative abundance of bacterial taxa annotated to OTUs at the phylum and genus level identified from the GIT digesta of piglets fed a control or XOS-supplemented diet. UC = unclassified at the phylum or genus level. CD = CON duodenum; X50D = XOS-50 duodenum; X500D = XOS-500 duodenum; CJ = CON jejunum; X50J = XOS-50 jejunum; X500J = XOS-500 jejunum; CI = CON ileum; X50I = XOS-50 ileum; X500I = XOS-500 ileum; CCa = CON caecum; X50Ca = XOS-50 caecum; X500Ca = XOS-500 caecum; CCo = CON colon; X50Co = XOS-50 colon; X500Co = XOS-500 colon; CR = CON rectum; X50R = XOS-50 rectum; X500R = XOS-500 rectum. Sample sizes for each day, GIT location, and diet group are as follows: d22—duodenum (CON: *n* = 10, XOS-50: *n* = 9, XOS-500: *n* = 9), jejunum (CON: *n* = 10, XOS-50: *n* = 10, XOS-500: *n* = 9), ileum (CON: *n* = 12, XOS-50: *n* = 12, XOS-500: *n* = 12), caecum (CON: *n* = 12, XOS-50: *n* = 12, XOS-500: *n* = 12), colon (CON: *n* = 12, XOS-50: *n* = 12, XOS-500: *n* = 12), and rectum (CON: *n* = 9, XOS-50: *n* = 12, XOS-500: *n* = 12); d54—duodenum (CON: *n* = 6, XOS-50: *n* = 9, XOS-500: *n* = 7), jejunum (CON: *n* = 6, XOS-50: *n* = 9, XOS-500: *n* = 7), ileum (CON: *n* = 11, XOS-50: *n* = 12, XOS-500: *n* = 11), caecum (CON: *n* = 12, XOS-50: *n* = 12, XOS-500: *n* = 12), colon (CON: *n* = 12, XOS-50: *n* = 12, XOS-500: *n* = 11), and rectum (CON: *n* = 12, XOS-50: *n* = 12, XOS-500: *n* = 11).

A total of twenty-two phyla were detected across all samples throughout the study. The most abundant phyla identified at day 22 were Firmicutes (69.79%), Bacteroidetes (22.44%), Proteobacteria (3.89%), and Spirochaetes (1.94%), while unclassified bacteria comprised 1.07% of the total sequences. The most abundant phyla identified at day 54 were Firmicutes (71.98%), Bacteroidetes (23.23%), Proteobacteria (1.51%), and Spirochaetes (1.25%), with unclassified bacteria accounting for 0.98% of the total sequences. A total of 4127 OTUs were identified across all samples throughout the study. The most abundant genera identified at day 22 were *Lactobacillus* (40.71%), *Prevotella* (16.74%), *Sarcina* (6.1%), *Megaspheara* (4.55%), *Clostridium sensu stricto* (4.1%), *Prevotellaceae* unclassified (3.67%), *Enterobacteriaceae* unclassified (2.68%), *Treponema* (1.94%), *Clostridium_XI* (1.63%), and *Lachnospiraceae* unclassified (1.55%). The most abundant genera identified at day 54 were *Lactobacillus* (35.34%), *Prevotella* (18.57%), *Clostridium_sensu_stricto* (8.7%), *Megaspheara* (4.74%), *Clostridium_XI* (4.04%), *Prevotellaceae* unclassified (3.31%), *Streptococcus* (2.6%), *Sarcina* (2.54%), *Lachnospiraceae* unclassified (1.68%), and *Veillonellaceae* unclassified (1.62%). Relative abundances of the top ten and top twenty bacterial taxa identified throughout the pig GIT at the phylum and genus level, respectively, at day 22 and 54, are shown in Figure 4.

**Figure 5:**
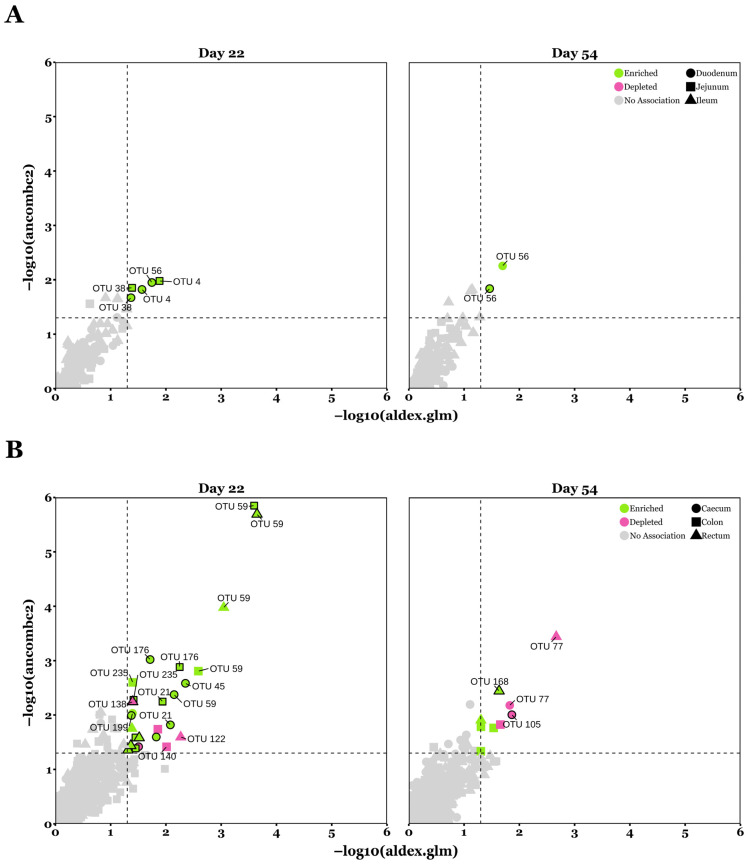
Discriminative taxa identified by ALDEx2 and ANCOM-BC2 between control and XOS-fed pigs. (**A**) Differentially abundant OTUs in the duodenum, jejunum, and ileum between control and XOS-fed pigs. (**B**) Differentially abundant OTUs in the caecum, colon, and rectum between control and XOS-fed pigs. Shapes without a black outline represent differentially abundant OTUs between CON and XOS-50 pigs; shapes with a black outline represent differentially abundant OTUs between CON and XOS-500 pigs. Sample sizes for each day, GIT location, and diet group are as follows: d22—duodenum (CON: *n* = 10, XOS-50: *n* = 9, XOS-500: *n* = 9), jejunum (CON: *n* = 10, XOS-50: *n* = 10, XOS-500: *n* = 9), ileum (CON: *n* = 12, XOS-50: *n* = 12, XOS-500: *n* = 12), caecum (CON: *n* = 12, XOS-50: *n* = 12, XOS-500: *n* = 12), colon (CON: *n* = 12, XOS-50: *n* = 12, XOS-500: *n* = 12), and rectum (CON: *n* = 9, XOS-50: *n* = 12, XOS-500: *n* = 12); d54—duodenum (CON: *n* = 6, XOS-50: *n* = 9, XOS-500: *n* = 7), jejunum (CON: *n* = 6, XOS-50: *n* = 9, XOS-500: *n* = 7), ileum (CON: *n* = 11, XOS-50: *n* = 12, XOS-500: *n* = 11), caecum (CON: *n* = 12, XOS-50: *n* = 12, XOS-500: *n* = 12), colon (CON: *n* = 12, XOS-50: *n* = 12, XOS-500: *n* = 11), and rectum (CON: *n* = 12, XOS-50: *n* = 12, XOS-500: *n* = 11). A list of bacterial taxa annotated to the OTUs presented in this figure is shown in Table 3.

ALDEx2 and ANCOM-BC2 were used to calculate significant differences in the differential abundance of OTUs assigned to bacterial taxa at the genus level with a minimum prevalence threshold of 10% and a minimum depth of 50 sequences (Figure 5).

In total, for the upper GIT and across both timepoints, seven OTUs were significantly more abundant (*p* < 0.05) in XOS-fed pigs according to both ALDEx2 and ANCOM-BC2, with five in duodenal and two in jejunal samples. Meanwhile, no OTUs in the small intestine were suppressed by XOS (*p* > 0.05). For the lower intestinal communities, twenty-six OTUs were significantly enriched overall by XOS (*p* < 0.05), with seven occurring in caecal, eleven in colonic, and eight in rectal samples. Comparatively, nine OTUs were significantly depleted in XOS-fed pigs (*p* < 0.05), with three occurring in caecal, three in colonic and three in rectal samples. A full list of taxa that were significantly modified by XOS according to both ALDEx2 and ANCOM-BC2, including *p*-values and effect sizes, is provided in Table 3. *Lactobacillus* (OTU 4, OTU 38, and OTU 56) was enriched in the upper GIT bacterial communities of XOS-fed pigs. For lower GIT communities, *Clostridium_XI* (OTU 59) was significantly enriched, while Veillonellaceae UC (OTU 77) was depleted, in XOS-fed pigs.

*Salmonella* V4 rRNA sequences were detected in the microbiome sequence data, prompting further investigation into the presence of *Salmonella* during the study.

### 3.5. Salmonella Quantification and Correlation with Alpha Diversity

**Figure 6:**
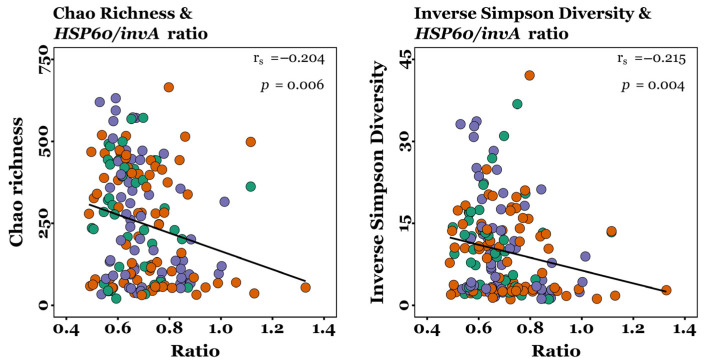
Correlations between alpha diversity and *HSP60/invA* ratios. Green points, CON (*n* = 49); orange points, XOS-50 (*n* = 73); purple points, XOS-500 (*n* = 68). r_s_, Spearman’s rank correlation coefficient; *p*, Spearman’s rank test *p*-value.

Digesta samples were incubated in BPW at 37 °C for 16 h, followed by plating on MSRV agar and incubation at 42 °C for 48 h. No *Salmonella* were isolated by enrichment from any of the samples. Since *Lactobacillus* was not differentially abundant between dietary groups for any timepoint or GIT site (*p* > 0.05; Kruskal–Wallis tests), qPCR was performed to quantify *Salmonella* by targeting *invA* and *Lactobacillus* by targeting *HSP60* in all samples. For each sample, ct values were used to calculate a *HSP60*/*invA* ratio. No significant differences in *HSP60*/*invA* ratios were observed between diet groups throughout the GIT for day 22 or 54 (*p* > 0.05; Kruskal–Wallis tests), demonstrating that *Salmonella* was not differentially abundant at any time. However, the *HSP60*/*invA* ratios were significantly correlated with alpha diversity indices across all samples. A weak, but statistically significant, negative correlation was observed between *HSP60*/*invA* ratios and Chao richness (r_s_ = −0.204 and *p* = 0.006) and between *HSP60*/*invA* ratios and inverse Simpson diversity (r_s_ = −0.215 and *p* = 0.004), as determined by Spearman’s rank correlation (Figure 6).

### 3.6. Histology and Gut Architecture

**Figure 7:**
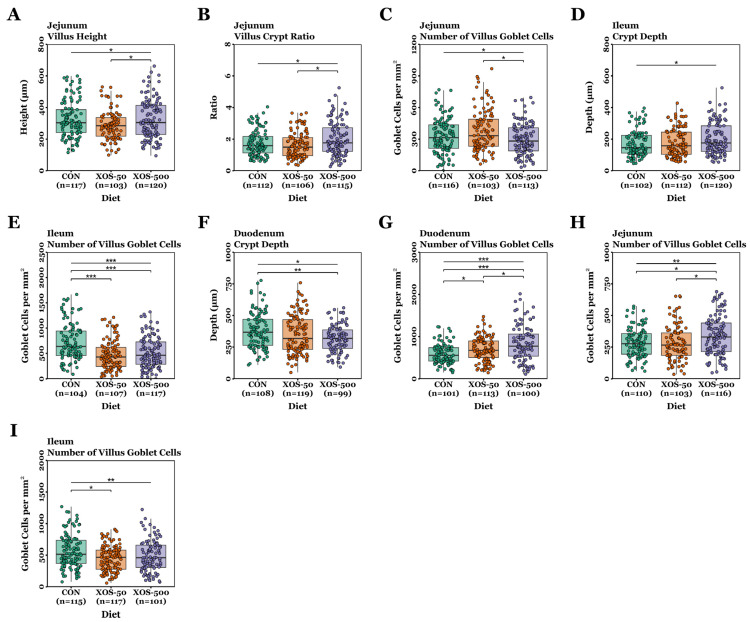
Differences in upper intestinal gut architecture and GC expression between control and XOS-fed pigs. Gut architecture and GC density changes on d22 in the jejunum (**A**–**C**) and ileum (**D**,**E**), and on d54 in the duodenum (**F**,**G**), jejunum (**H**), and ileum (**I**). *, *p* < 0.05; **, *p* < 0.01; ***, *p* < 0.001; Kruskal–Wallis and Dunn’s tests.

Villus height, crypt depth, VCR, and GC density measurements were not normally distributed (*p* < 0.05, Shapiro–Wilk tests) and were, therefore, analysed using non-parametric Kruskal–Wallis tests, followed by Dunn’s test for pairwise comparisons. The histology results are presented as boxplots in Figure 7.

On day 22, no significant differences in crypt GC density were detected between diet groups across the duodenum, jejunum, and ileum (*p* > 0.05). Jejunal villus height and VCR were significantly greater in the XOS-500 diet group compared to the XOS-50 group (*p* = 0.04 and *p* = 0.021, respectively). The Kruskal–Wallis test revealed a significant difference in ileal crypt depth (*p* = 0.044); however, pairwise comparisons revealed no significant difference between diet groups (*p* > 0.05). Villus GC density in the jejunum was greater in pigs fed the XOS-50 diet compared to those receiving the XOS-500 diet (*p* = 0.011). In the ileum, both the XOS-50 and XOS-500 groups showed significantly reduced villus GC density compared to the control group (*p* = 2.55 × 10^−7^ and *p* = 1.93 × 10^−5^, respectively).

On day 54, no significant differences were detected in villus height, VCR, or crypt GC density among the diet groups across the duodenum, jejunum, and ileum (*p* > 0.05). However, duodenal crypt depth was significantly lower in XOS-500 pigs compared to controls (*p* = 0.009). Duodenal villus GC density increased significantly as the concentration of XOS in the diet increased; GC density was significantly greater in XOS-50 pigs compared to controls (*p* = 0.015), while GC density was significantly greater in XOS-500 compared to the XOS-50 group (*p* = 0.028). In the jejunum, GC density was significantly greater in XOS-500 pigs compared to controls and XOS-50 pigs (*p* = 0.02 and *p* = 0.012, respectively). Ileal GC density was significantly lower in XOS-50 pigs compared to controls (*p* = 0.009).

### 3.7. Immunomodulation

Fold change measurements were normally distributed for most samples (Shapiro–Wilk tests *p* > 0.05), but not all. No significant differences in the mRNA expression levels of *OCLN*, *ZO-2*, *IL-1β*, *IL6*, and *IL10* were observed between the control, XOS-50 and XOS-500 diet groups across the GIT throughout the study (Kruskal–Wallis tests, *p* > 0.05).

**Figure 8:**
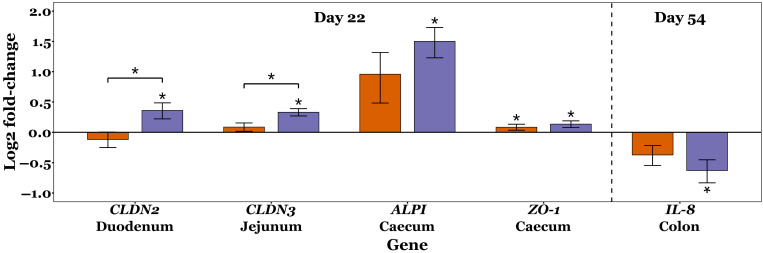
Changes in expression of *CLDN2*, *CLDN3*, *ALPI*, *ZO-1*, and *IL-8* in piglet GIT tissues. Gene expression was quantified by qRT-PCR, and fold change was calculated using the 2^−ΔΔct^ method. Fold changes were then log_2_-transformed, where all CON values are zero by definition and, therefore, are omitted from the plot, allowing upregulation and downregulation to be visualised as deviations from the *x*-axis. Expression of each gene of interest was normalised to the average of housekeeping genes GAPDH and RPL4 for each sample. Orange bars represent XOS-50 log_2_ (fold changes), and purple bars represent XOS-500 log_2_ (fold changes). An asterisk directly above or below a bar indicates a significant difference compared to the CON group, while an asterisk with a connecting line between bars denotes a significant difference between XOS-50 and XOS-500 pigs. For each sample and gene of interest, ct values >35 were excluded from analysis, and outliers were removed using the 1.5 × IQR method. Sample sizes for each gene and diet group presented are as follows: *CLDN2* (CON: *n* = 10, XOS-50: *n* = 11, XOS-500: *n* = 10), *CLDN3* (CON: *n* = 11, XOS-50: *n* = 12, XOS-500: *n* = 11), *ALPI* (CON: *n* = 9, XOS-50: *n* = 11, XOS-500: *n* = 9), *ZO-1* (CON: *n* = 9, XOS-50: *n* = 10, XOS-500: *n* = 11), and *IL-8* (CON: *n* = 11, XOS-50: *n* = 11, XOS-500: *n* = 11). *, *p* < 0.05.

On day 22, *CLDN2* expression in the duodenum was significantly upregulated in XOS-500 pigs compared to both control and XOS-50 pigs (*p* = 0.042 and *p* = 0.025, respectively; Dunn’s test). Similarly, *CLDN3* expression in the jejunum was significantly greater in XOS-500 pigs compared to control and XOS-50 groups (*p* = 0.02 and *p* = 0.041, respectively). In the caecum, *ALPI* expression was significantly increased in XOS-500 pigs compared to controls (*p* = 0.021), while *ZO-1* expression was greater in both XOS-50 and XOS-500 pigs compared to controls (*p* = 0.033 and *p* = 0.015, respectively). On day 54, colonic *IL-8* expression was significantly lower in XOS-500 pigs compared to controls (*p* = 0.026). The effects of XOS on target gene expression is shown in Figure 8.

## 4. Discussion

The objectives of this study were to investigate the effects of XOS supplementation on performance, gut microbiome composition, immunomodulation, gut barrier integrity, and gut morphology in weaning piglets naturally exhibiting PWD, a condition commonly observed during commercial pig production.

In the present study, pigs supplemented with 500 mg of XOS per kg of feed for 22 and 54 days showed a significant increase in body weight of 10.8 and 7.4%, respectively, while supplementation with 50 mg/kg did not significantly improve growth performance compared to controls (Figure 1). Although ADG tended to increase with the concentration of in-feed XOS, and FCR showed a tendency toward greater efficiency in the XOS-500 diet group, neither effect was statistically significant (Figure 2). It is possible that the lack of statistical significance in ADG was due to the reduced sensitivity of the ANOVA across the full study period (d1–d54), given that no significant differences in BW were observed at d1, d7, or d14, but were observed at d22 and d54. Additionally, DFI was not significantly different between diet groups from d1–d54 (Figure 2), demonstrating that increased weight gain was not due to increased gross calorie intake. Rather, the observed increase in BW may reflect improved nutrient digestibility, as observed in other studies where XOS enhanced the apparent total tract digestibility of dry matter, nitrogen, and gross energy in weaning pigs [21]. Previous studies have shown that healthy 28-day-old XOS-fed piglets displayed a 4.7% increase in body weight compared to control animals in a 28-day study, while ADG improved by 9.6% and gain-to-feed ratio (G:F) improved by 7.1% [20]. Similarly, healthy piglets weaned at 30 da and supplemented with XOS for 28 days displayed an 8.6% increase in body weight and a 15.5% increase in ADG, but showed no significant difference in G:F [22]. In comparison, XOS-fed piglets in this study achieved a greater increase in body weight despite receiving lower concentrations of in-feed XOS. However, unlike previous studies that examined healthy piglets, those in the present study were experiencing PWD. This suggests that XOS supplementation may have a more pronounced effect on growth performance in piglets suffering from naturally occurring PWD compared to healthy subjects.

There were no significant differences in α-diversity throughout the GIT between control and XOS-fed pigs in this study (Table 1 and Figure S2). In comparison, it is reported that supplementing the diets of healthy post-weaning piglets with XOS significantly increases the Chao1 and Shannon indices of ileal and caecal communities [18]. Additionally, XOS significantly increased Chao1 and ACE indices, but had no effect on Shannon or Simpson indices, for the distal gut communities of healthy pigs [50]. However, there were no significant differences in species richness (ACE and Chao1) or evenness (Shannon and inverse Simpson) reported for caecal communities in lipopolysaccharide (LPS)-challenged piglets fed XOS compared with controls [23]. Although no significant differences in α-diversity were observed between dietary groups in this study, Chao richness and inverse Simpson diversity significantly increased from the duodenum to the rectum on both day 22 and 54 in control and XOS-fed pigs (Table 1 and Figure S2), demonstrating the establishment of more diverse microbial communities in the lower GIT compared to the upper, consistent with previous work [51]. Greater alpha diversity has been linked with greater body weight in pigs. Han et al. (2017) [52] reported that heavier piglets exhibited significantly greater ACE and Chao1 indices and more OTUs than lighter counterparts, despite being genetically identical and reared under uniform conditions. Similarly, in this study, jejunal Chao richness positively correlated with final body weight (Figure 3A), aligning with observations in healthy pre-weaned piglets, where Chao1 positively correlated with pre-wean weight gain [53]. Moreover, newborn piglets with intrauterine growth restriction have been found to show lower jejunal Chao and Shannon alpha diversity indices, compared to normal birthweight piglets [54]. In this study, alpha diversity expressed as Chao richness and inverse Simpson diversity was weakly negatively correlated with *HSP60*/*invA* ratios across all samples (Figure 6), suggesting that more diverse microbial communities may enhance colonisation resistance against enteric pathogens, consistent with previous findings [55]. Although *Salmonella* DNA was detected in digesta samples, it is important to acknowledge that the PWD observed in this study may have been caused by other common enteric pathogens, such as enterotoxigenic *E. Coli* and rotavirus, which were not investigated in this study. *Salmonella* was pursued further based on its unexpected presence in the raw MiSeq sequencing output, which prompted targeted confirmation using qPCR assays. While detection of the *invA* gene indicates the presence of *Salmonella* species with the genetic potential for epithelial invasion, *invA* is conserved across both pathogenic and non-pathogenic serovars; therefore, its presence alone does not confirm active infection or pathogenicity in pigs [56]. Nevertheless, our findings demonstrate that greater alpha diversity may contribute to reduced relative abundance of *Salmonella* spp., supporting a potential role of XOS in promoting colonisation resistance under commercial pig production settings.

AMOVA of jejunal communities on day 22 revealed a significant difference in beta diversity between the control and XOS-500 groups, as indicated by the Yue and Clayton and Bray–Curtis dissimilarity indices (Table 2 and Figure S3). In contrast, AMOVA of the Jaccard similarity index showed no significant difference between any diet groups throughout the study, suggesting that supplementation with 500 mg/kg transiently influences microbial community structure rather than overall community membership. Previous studies have reported that the β-diversity of GIT microbial communities of healthy XOS-fed pigs differ from that of control groups in the ileum [7,14] and caecum [18]. However, these studies do not report statistical analyses, such as AMOVA. Nonetheless, their findings indicate that XOS supplementation modulates GIT microbial composition.

A total of 22 phyla were detected across all pig GIT microbial communities throughout the study, comprising 4127 OTUs. Of these twenty-two phyla, Firmicutes dominated the small intestine, while the large intestine was co-dominated by Firmicutes and Bacteroidetes, in keeping with previous results [51,57,58]. Firmicutes was the most abundant phylum in the small intestine, primarily due to the high abundance of the genus *Lactobacillus* (Figure 4). Lactobacilli are able to colonise the acidic environment of the small intestine, owing to their resistance to low pH [59], which is caused by the secretion of bile acids from the gallbladder into the duodenal lumen [60]. Moreover, members of the Firmicutes phylum encode carbohydrate transport phosphotransferase systems [61], which facilitate the absorption of simple molecules from the small intestinal lumen. The small intestine metagenome lacks the ability to metabolise plant cell wall (PCW) material [62], a major component of pig feed, resulting in the incomplete digestion of complex carbohydrates. Consequently, this undigested material reaches the large intestine where it is anaerobically fermented by members of the Bacteroidetes phylum, which possess a diverse range of enzymes for PCW degradation [63]. Among these is the genus *Prevotella*, which accounted for 29–43% of the relative abundance of OTUs in the large intestine across all samples in this study, compared with just 0.1–4.2% in the small intestine (Figure 4). Differentially abundant bacterial taxa at the genus level were identified between diet groups using ALDEx2 and ANCOM-BC2 (Figure 5). Dietary XOS supplementation had a greater impact on microbiota composition in the large intestine compared to the small intestine, with thirty-five taxa exhibiting significant changes in abundance in the hindgut, compared to seven in the foregut. Similarly, the effect of XOS was more pronounced on day 22 than on day 54, as indicated by thirty-one differentially abundant taxa on day 22 compared to eleven on day 54. Notably, only a single OTU, *Lactobacillus* (OTU 56), retained its differential abundance across both timepoints, suggesting a limited legacy effect of XOS. This may indicate that XOS exerts its effects on the microbiome early, driving the enrichment or depletion of taxa within 22 days, after which microbial communities converge over time, despite continued supplementation with XOS, consistent with the β-diversity observations in this study. In the small intestine, *Lactobacillus* was the only genus that was differentially abundant in response to XOS supplementation. This is consistent with previous studies on healthy post-weaning piglets of a similar age, which demonstrated increased ileal populations of *Lactobacillus* following XOS supplementation [16,18]. Additionally, XOS has been shown to stimulate *Lactobacillus* growth in the caecum [18,64] and faeces [21,22] of weaning piglets and in the colon of fattening pigs [65]. *Lactobacillus* contributes to the modulation of the GIT lumen environment, through short-chain fatty acid (SCFA) synthesis and lowering the pH, thereby reducing colonisation by pathogens, increasing energy availability to enterocytes, supporting gut barrier function, and exerting anti-inflammatory effects [66]. Although no bifidogenic effects were observed in this study, XOS has been shown to increase *Bifodbacterium* abundance in vitro and in weaning pigs [15,16,17]. In a *Salmonella* Typhimurium challenge model in mice, XOS significantly increased the relative abundance of colonic *Bifidobacterium animalis*, inhibited *Salmonella* colonisation, and reduced *Salmonella* translocation to the liver and spleen. This was associated with decreased colonic expression of pro-inflammatory *CXCL-1* which contributes to increased gut permeability [25]. The abundance of *Clostridium_XI* was significantly increased throughout the large intestine in both the XOS-50 and XOS-500 groups. Previous studies have shown that *Clostridium_XI* is positively correlated with body weight and average daily gain in commercially bred hybrid pigs [67], and that it is more prevalent in low residual feed intake pigs [68], indicating a potential role in improved feed conversion efficiency. Additionally, *Clostridium_XI* has been associated with increased SCFA production when pre-weaned piglets are fed a commercial milk replacer alongside suckling [69]. Similarly, increased populations of *Clostridium_XI* has been observed in the jejunal microbiome of suckling lambs compared to bottle-fed lambs [70], indicating that this bacterial cluster may contribute to early gut development. *Veillonellaceae* UC (OTU 77) was significantly downregulated across the caecum, colon, and rectum of pigs in the XOS-50 group compared to controls. Previous studies have reported increased *Veillonellaceae* populations in patients with active ulcerative colitis, where their abundance was positively correlated with the pro-inflammatory chemokines CXCL16 and IL-8 [71]. Similarly, increased *Veillonellaceae* abundance is observed in piglets subjected to oxidative stress, where chronic oxidative stress induced by D-galactose modulated the gut microbiota and led to a significant increase in *Veillonellaceae* [72]. These findings suggest that *Veillonellaceae* may proliferate in inflammatory conditions, and their downregulation in response to XOS supplementation in this study may indicate a shift toward a less inflammatory gut environment.

Despite variations in gut morphological responses, this study demonstrates that XOS supplementation significantly modulates gut architecture and GC expression in weaning piglets (Figure 7). On day 22, pigs in the XOS-50 and XOS-500 groups displayed a significantly lower number of villus GCs in the ileum compared to controls. However, by day 54, duodenal villus GC numbers increased progressively with XOS concentration, while there were significantly more villus GCs in the jejunum of XOS-500 pigs compared to controls. Additionally, by day 54, XOS-500 pigs exhibited significantly reduced crypt depth compared to controls. These findings suggest that XOS may transiently alter gut morphology and GC expression in the early post-weaning period but promote greater mucosal adaptation over time, potentially through enhanced microbial fermentation. In particular, SCFA-producing bacteria, such as *Clostridium_XI* and *Lactobacillus* spp., which were significantly increased in this study by XOS, may support mucosal regeneration through lactic-acid mediated stimulation of intestinal stem, Paneth, and goblet cells [73]. These findings are in keeping with previous studies demonstrating that XOS increases villus height and VCR in healthy weaning pigs [17,18,20]. Furthermore, XOS supplementation has been shown to enhance gut morphology, particularly by increasing villus height and optimising crypt depth, in pigs challenged with *E. coli* LPS [23,24], indicating its potential role in supporting intestinal integrity under both normal and inflammatory conditions.

XOS supplementation influenced the expression of mRNA encoding key tight junction proteins (TJPs), with *CLDN2* and *CLDN3* significantly upregulated in the XOS-500 group, while *ZO-1* expression was significantly increased in both XOS-50 and XOS-500 groups, compared to controls on day 22 (Figure 8). Tight junction proteins are essential for maintaining intestinal barrier integrity by regulating paracellular permeability and preventing the translocation of harmful bacteria and their associated toxins from the gut lumen to the rest of the body. Claudin-2 forms selective paracellular channels that increase permeability to small molecules and water, whereas claudin-3 reinforces barrier integrity by sealing tight junctions. Although previous studies have reported no significant change in *CLDN2* or *CLDN3* expression in XOS-supplemented weaning pigs [17,20,74], both were upregulated in this study, consistent with findings in broilers supplemented with XOS [75]. The upregulation of *ZO-1*, a scaffolding protein that links transmembrane tight junction components, such as claudins, to the actin cytoskeleton to provide structural stability and maintain barrier function, aligns with previous studies that consistently report that XOS upregulates *ZO-1* expression in pigs [17,20,50,64]. The observed increase in caecal *ALPI* expression in XOS-500 pigs may have contributed to the upregulation of TJPs, as ALPI plays a critical role in maintaining intestinal barrier integrity. ALPI-mediated dephosphorylation of the lipid A moiety of bacterial LPS reduces the activation of Toll-like receptor 4 (TLR4) and downstream NF-κB signalling [76]. This inhibition of NF-κB attenuates inflammatory responses that would otherwise compromise gut barrier function and increase gut permeability, potentially leading to antigen translocation [77]. ALPI activity is stimulated by butyrate, an SCFA produced by fibre-fermenting bacteria, with *Lactobacillus*—which was increased in this study by XOS—enhancing butyrate production in the gut through lactate and acetate synthesis [78,79,80]. Additionally, previous studies have demonstrated that *ALPI*-knockout mice exhibit decreased *ZO-1* and *ZO-2* expression, further supporting the role of ALPI in regulating tight junction integrity [81]. *ALPI* expression reportedly decreases in piglets following early weaning compared to those that continue to suckle, which may contribute to increased intestinal permeability and inflammatory responses observed post-weaning [82]. This is further exacerbated by the depletion of beneficial lactic acid bacteria, such as *Lactobacillus*, while the inflammatory cytokines IL-1β, IL-8, IL-6, and TNF-α are upregulated post-weaning [6,7]. The significant suppression of colonic *IL-8* in XOS-500 pigs on day 54 may be a consequence of increased *ALPI* expression earlier in the study. NF-κB is a key regulator of IL-8 transcription in intestinal epithelial cells, and its activity may have been modulated by reduced TLR4 activation, contributing to the observed reduction in *IL-8* expression [77]. This indicates a dampened inflammatory response and improved gut barrier function, consistent with ALPI’s role in mitigating LPS-induced inflammation and maintaining intestinal homeostasis.

## 5. Conclusions

We have demonstrated that dietary supplementation of 28-day old weaning piglets with 500 mg of XOS per kg of feed significantly improves growth performance in pigs suffering from naturally occurring PWD. Additionally, we observed a significant positive correlation between alpha diversity and piglet body weight, and a significant negative correlation between Chao richness and DFI, suggesting that a more diverse microbiota may enhance feed conversion efficiency. Moreover, greater microbial diversity was weakly negatively correlated with *Salmonella* presence in the gut, suggesting a potential association with pathogen inhibition and colonisation resistance, although the specific species composition and microbial functions likely play key roles. Supplementation with XOS modulated gut microbiota composition, with an increase in *Lactobacillus*, a lactic acid bacterium known to enhance SCFA production through lactate and acetate synthesis. This may have contributed to improved gut health via SCFA-mediated effects on barrier function and immune regulation. Dietary XOS had a transient effect on gut morphology and GC expression, with reduced crypt depth and a greater number of GCs observed in XOS-fed piglets observed by 54 days of supplementation. Improvements in gut barrier integrity coincided with the increased expression of tight junction protein genes and *ALPI*, a brush border enzyme that dephosphorylates bacterial LPS, reducing TLR4 activation, NF-κB signalling, and gut permeability. Furthermore, XOS suppressed *IL-8*, potentially due to increased *ALPI* expression and its role in modulating inflammatory signalling.

In summary, we found that XOS supplemented at 500 mg per kg of feed is efficacious in improving growth performance and gut health in weaning piglets, despite the presence of naturally occurring PWD, reflecting conditions typical of a commercial production setting.

## Figures and Tables

**Table 1:** Alpha diversity. Mean (SD).

GIT Site		Day 22			Day 54	
	CON	XOS-50	XOS-500	CON	XOS-50	XOS-500
	Chao Richness					
Duodenum	120.4 (60.56)	120.07 (78.29)	96.43 (53.01)	150.24 (98.24)	145.44 (75.94)	148.5 (75.64)
Jejunum	78.95 (36.69)	63.92 (18.05)	89.46 (33.8)	133.03 (53.88)	95.13 (29.13)	98.83 (47.31)
Ileum	56.32 (29.86)	61.21 (25.95)	92.49 (57.19)	101.73 (68.47)	98.38 (57.99)	81.45 (35.06)
Caecum	281.58 (66.47)	287.55 (79.46)	271.58 (84.37)	264.18 (68.02)	219.46 (81.38)	259.73 (75.68)
Colon	388.29 (85.51)	408.23 (97.15)	396.54 (81.6)	405.49 (88.77)	394.5 (71.75)	402.11 (59.57)
Rectum	454.51 (89.14)	462.56 (80.43)	450.28 (75.07)	469.2 (66.13)	423.5 (92.73)	447.84 (80.98)
	Inverse Simpson diversity					
Duodenum	2.61 (1.51)	3.03 (2.3)	2.37 (0.76)	3.51 (2.06)	4.21 (2.23)	5.8 (4.31)
Jejunum	2.16 (0.48)	2.62 (0.92)	2.26 (0.65)	4.86 (4.24)	3.23 (0.75)	3.51 (2.08)
Ileum	2.99 (1.19)	3.3 (1.08)	3.38 (0.91)	3.57 (1.51)	3.33 (1.04)	3.11 (1.05)
Caecum	13.15 (5.3)	13.67 (4.43)	13.15 (6.52)	13.36 (5.54)	11.31 (5.36)	13.81 (4)
Colon	15.14 (6.35)	14.95 (5.7)	12.28 (2.42)	14.03 (5.62)	15.35 (7.1)	16.21 (5.02)
Rectum	17.74 (9.14)	17.57 (7.57)	13.21 (4.23)	20.31 (9.76)	18.1 (8.16)	19.91 (7.4)

Sample sizes for each day, GIT location, and diet group are as follows: d22—duodenum (CON: *n* = 10, XOS-50: *n* = 9, XOS-500: *n* = 9), jejunum (CON: *n* = 10, XOS-50: *n* = 10, XOS-500: *n* = 9), ileum (CON: *n* = 12, XOS-50: *n* = 12, XOS-500: *n* = 12), caecum (CON: *n* = 12, XOS-50: *n* = 12, XOS-500: *n* = 12), colon (CON: *n* = 12, XOS-50: *n* = 12, XOS-500: *n* = 12), and rectum (CON: *n* = 9, XOS-50: *n* = 12, XOS-500: *n* = 12); d54—duodenum (CON: *n* = 6, XOS-50: *n* = 9, XOS-500: *n* = 7), jejunum (CON: *n* = 6, XOS-50: *n* = 9, XOS-500: *n* = 7), ileum (CON: *n* = 11, XOS-50: *n* = 12, XOS-500: *n* = 11), caecum (CON: *n* = 12, XOS-50: *n* = 12, XOS-500: *n* = 12), colon (CON: *n* = 12, XOS-50: *n* = 12, XOS-500: *n* = 11), and rectum (CON: *n* = 12, XOS-50: *n* = 12, XOS-500: *n* = 11).

**Table 2:** Beta diversity showing significant differences in AMOVA between CON and XOS-fed pigs.

GIT Site		Day 22			Day 54	
	CON—XOS-50	CON—XOS-500	XOS-50—XOS-500	CON—XOS-50	CON-XOS-500	XOS-50—XOS-500
	Yue and Clayton Dissimilarity					
Duodenum	0.938	0.079	0.247	0.833	0.811	0.479
Jejunum	0.687	0.005	0.100	0.974	0.640	0.357
Ileum	0.870	0.091	0.213	0.376	0.265	0.932
Caecum	1.000	0.268	0.184	0.896	0.809	0.299
Colon	0.493	0.255	0.409	0.309	0.555	0.960
Rectum	0.054	0.141	0.059	0.508	0.729	0.786
	Bray–Curtis Dissimilarity					
Duodenum	0.854	0.086	0.314	0.836	0.950	0.564
Jejunum	0.689	0.004	0.134	0.941	0.518	0.344
Ileum	0.738	0.123	0.342	0.338	0.250	0.919
Caecum	0.973	0.348	0.428	0.853	0.936	0.380
Colon	0.602	0.318	0.350	0.529	0.887	0.994
Rectum	0.173	0.187	0.051	0.487	0.656	0.990
	Jaccard Similarity					
Duodenum	0.345	0.509	0.526	0.857	0.929	0.919
Jejunum	0.374	0.324	0.125	0.309	0.106	0.659
Ileum	0.088	0.228	0.219	0.543	0.290	0.611
Caecum	0.336	0.270	0.341	0.988	0.190	0.147
Colon	0.260	0.086	0.769	0.486	0.546	0.733
Rectum	0.358	0.158	0.808	0.443	0.328	0.707

Sample sizes for each day, GIT location, and diet group are as follows: d22—duodenum (CON: *n* = 10, XOS-50: *n* = 9, XOS-500: *n* = 9), jejunum (CON: *n* = 10, XOS-50: *n* = 10, XOS-500: *n* = 9), ileum (CON: *n* = 12, XOS-50: *n* = 12, XOS-500: *n* = 12), caecum (CON: *n* = 12, XOS-50: *n* = 12, XOS-500: *n* = 12), colon (CON: *n* = 12, XOS-50: *n* = 12, XOS-500: *n* = 12), and rectum (CON: *n* = 9, XOS-50: *n* = 12, XOS-500: *n* = 12); d54—duodenum (CON: *n* = 6, XOS-50: *n* = 9, XOS-500: *n* = 7), jejunum (CON: *n* = 6, XOS-50: *n* = 9, XOS-500: *n* = 7), ileum (CON: *n* = 11, XOS-50: *n* = 12, XOS-500: *n* = 11), caecum (CON: *n* = 12, XOS-50: *n* = 12, XOS-500: *n* = 12), colon (CON: *n* = 12, XOS-50: *n* = 12, XOS-500: *n* = 11), and rectum (CON: *n* = 12, XOS-50: *n* = 12, XOS-500: *n* = 11).

**Table 3:** Bacterial taxa annotated to OTUs that were differentially abundant between control and XOS-fed pigs according to ALDEx2 and ANCOM-BC2.

Day	Taxon	ALDEx2		ANCOM-BC2		GIT Site
		Effect	*p*-Value	Effect	*p*-Value	
22	^2^ *Lactobacillus* (OTU 4)	0.568	0.027	2.348	0.015	Duodenum
	^2^ *Lactobacillus* (OTU 38)	0.536	0.043	2.458	0.021	
	^2^ *Lactobacillus* (OTU 56)	0.639	0.018	2.292	0.011	
	^2^ *Lactobacillus* (OTU 4)	0.855	0.013	2.679	0.011	Jejunum
	^2^ *Lactobacillus* (OTU 38)	0.589	0.041	2.749	0.014	
	^2^ *Acidaminococcus* (OTU 18)	−0.484	0.031	−1.055	0.038	Caecum
	^2^ Prevotellaceae UC (OTU 21)	0.564	0.008	2.360	0.015	
	^2^ *Prevotella* (OTU 45)	0.559	0.004	1.216	0.003	
	^2^ *Clostridium_XI* (OTU 59)	0.550	0.007	1.468	0.004	
	^2^ Bacteroidales UC (OTU 86)	0.516	0.015	0.894	0.025	
	^2^ Lachnospiraceae UC (OTU 176)	0.757	0.019	1.754	0.001	
	^2^ *Succinivibrio* (OTU 185)	0.526	0.042	1.428	0.010	
	^1^ *Prevotella* (OTU 199)	0.650	0.040	1.541	0.009	
	^2^ *Roseburia* (OTU 15)	0.463	0.035	0.929	0.041	Colon
	^2^ Prevotellaceae UC (OTU 21)	0.532	0.012	2.828	0.006	
	^2^ *Clostridium_XI* (OTU 59)	0.607	<0.001	2.216	<0.001	
	^2^ Bacteroidales UC (OTU 86)	0.438	0.035	0.948	0.026	
	^2^ Lachnospiraceae UC (OTU 176)	0.880	0.006	1.649	0.001	
	^2^ *Succinivibrio* (OTU 235)	0.249	0.039	1.180	0.005	
	^1^ *Clostridium_XI* (OTU 59)	0.133	0.003	1.449	0.002	
	^1^ Ruminococcaceae UC (OTU 122)	−0.441	0.014	−1.109	0.018	
	^1^ Ruminococcaceae UC (OTU 140)	−0.346	0.010	−0.944	0.038	
	^1^ *Succinivibrio* (OTU 235)	0.375	0.040	1.273	0.003	
	^2^ Prevotellaceae UC (OTU 21)	0.486	0.048	1.833	0.044	Rectum
	^2^ *Clostridium_XI* (OTU 59)	0.488	<0.001	1.952	<0.001	
	^2^ *Treponema* (OTU 138)	−0.590	0.040	−2.137	0.006	
	^2^ Lachnospiraceae UC (OTU 144)	0.310	0.030	0.988	0.026	
	^2^ Lachnospiraceae UC (OTU 183)	0.485	0.043	1.394	0.037	
	^1^ *Clostridium_XI* (OTU 59)	0.189	0.001	1.591	<0.001	
	^1^ Ruminococcaceae UC (OTU 122)	−0.656	0.005	−1.006	0.025	
	^1^ Ruminococcaceae UC (OTU 272)	0.598	0.041	1.213	0.018	
54	^2^ *Lactobacillus* (OTU 56)	0.162	0.034	2.037	0.015	Duodenum
	^1^ *Lactobacillus* (OTU 56)	0.339	0.020	2.189	0.006	
	^2^ *Peptococcus* (OTU 105)	−0.515	0.014	−1.391	0.010	Caecum
	^1^ Veillonellaceae UC (OTU 77)	−0.553	0.015	−2.006	0.007	
	^1^ Prevotellaceae UC (OTU 66)	0.352	0.029	0.818	0.017	Colon
	^1^ Veillonellaceae UC (OTU 77)	−0.407	0.022	−1.665	0.015	
	^1^ Lachnospiraceae UC (OTU 125)	0.098	0.050	1.043	0.046	
	^1^ Ruminococcaceae UC (OTU 142)	0.641	0.049	1.271	0.016	
	^2^ Firmicutes UC (OTU 168)	0.352	0.023	1.322	0.004	Rectum
	^1^ Veillonellaceae UC (OTU 77)	−0.646	0.002	−2.270	<0.001	
	^1^ Firmicutes UC (OTU 168)	0.134	0.049	1.110	0.012	

^1^ Taxa discriminative between CON and XOS-50 pigs. ^2^ Taxa discriminative between CON and XOS-500 pigs. UC = unclassified at the genus level. Sample sizes for each day, GIT location, and diet group are as follows: d22—duodenum (CON: *n* = 10, XOS-50: *n* = 9, XOS-500: *n* = 9), jejunum (CON: *n* = 10, XOS-50: *n* = 10, XOS-500: *n* = 9), ileum (CON: *n* = 12, XOS-50: *n* = 12, XOS-500: *n* = 12), caecum (CON: *n* = 12, XOS-50: *n* = 12, XOS-500: *n* = 12), colon (CON: *n* = 12, XOS-50: *n* = 12, XOS-500: *n* = 12), and rectum (CON: *n* = 9, XOS-50: *n* = 12, XOS-500: *n* = 12); d54—duodenum (CON: *n* = 6, XOS-50: *n* = 9, XOS-500: *n* = 7), jejunum (CON: *n* = 6, XOS-50: *n* = 9, XOS-500: *n* = 7), ileum (CON: *n* = 11, XOS-50: *n* = 12, XOS-500: *n* = 11), caecum (CON: *n* = 12, XOS-50: *n* = 12, XOS-500: *n* = 12), colon (CON: *n* = 12, XOS-50: *n* = 12, XOS-500: *n* = 11), and rectum (CON: *n* = 12, XOS-50: *n* = 12, XOS-500: *n* = 11).

## Data Availability

The data presented in this study are openly available at https://github.com/J-S-Stanley/XOS-2025 (accessed on 25 March 2025), with sequence data deposited in the NCBI database within the BioProject PRJNA1240184, with SRA records available at https://www.ncbi.nlm.nih.gov/sra/PRJNA1240184 (accessed on 22 March 2025).

## Supplementary Materials

The following supporting information can be downloaded at: https://www.mdpi.com/article/10.3390/microorganisms13081760/s1, Figure S1: Sequencing effort for all communities. Figure S2: Boxplots showing α-diversity throughout the GIT of pigs fed control or XOS-supplemented diets. Figure S3: NMDS showing β-diversity. Table S1: CON and XOS feed formulations. Table S2: Nutritional composition of pre-weaning creep feed offered to piglets from 15da to 28da. Table S3: Primer sequences for invA and HSP60 genes.

## References

[1] OECD, Food and Agriculture Organization of the United Nations (2021). OECD-FAO Agricultural Outlook 2021–2030.

[2] Dividich J.L., Rooke J.A., Herpin P. (2005). Nutritional and Immunological Importance of Colostrum for the New-Born Pig. J. Agric. Sci..

[3] Gormley A., Garavito-Duarte Y., Kim S.W. (2024). The Role of Milk Oligosaccharides in Enhancing Intestinal Microbiota, Intestinal Integrity, and Immune Function in Pigs: A Comparative Review. Biology.

[4] Moeser A.J., Ryan K.A., Nighot P.K., Blikslager A.T. (2007). Gastrointestinal Dysfunction Induced by Early Weaning Is Attenuated by Delayed Weaning and Mast Cell Blockade in Pigs. Am. J. Physiol. Gastrointest. Liver Physiol..

[5] Lallès J.-P., Boudry G., Favier C., Floc’h N.L., Luron I., Montagne L., Oswald I.P., Pié S., Piel C., Sève B. (2004). Gut Function and Dysfunction in Young Pigs: Physiology. Anim. Res..

[6] Gresse R., Chaucheyras-Durand F., Fleury M.A., Van de Wiele T., Forano E., Blanquet-Diot S. (2017). Gut Microbiota Dysbiosis in Postweaning Piglets: Understanding the Keys to Health. Trends Microbiol..

[7] Pié S., Lallès J.P., Sève B., Blazy F., Laffitte J., Oswald I.P. (2004). Weaning Is Associated with an Upregulation of Expression of Inflammatory Cytokines in the Intestine of Piglets. J. Nutr..

[8] Lallès J.-P., Bosi P., Smidt H., Stokes C.R. (2007). Nutritional Management of Gut Health in Pigs around Weaning. Proc. Nutr. Soc..

[9] Cromwell G.L. (2002). Why and How Antibiotics Are Used in Swine Production. Anim. Biotechnol..

[10] Han Y., Zhan T., Tang C., Zhao Q., Dansou D.M., Yu Y., Barbosa F.F., Zhang J. (2021). Effect of Replacing In-Feed Antibiotic Growth Promoters with a Combination of Egg Immunoglobulins and Phytomolecules on the Performance, Serum Immunity, and Intestinal Health of Weaned Pigs Challenged with Escherichia Coli K88. Animals.

[11] EC (2003). Regulation (EC) No 1831/2003 of the European Parliament and of the Council of 22 September 2003 on Additives for Use in Animal Nutrition.

[12] Gibson G.R., Roberfroid M.B. (1995). Dietary Modulation of the Human Colonic Microbiota: Introducing the Concept of Prebiotics. J. Nutr..

[13] Gibson G.R., Hutkins R., Sanders M.E., Prescott S.L., Reimer R.A., Salminen S.J., Scott K., Stanton C., Swanson K.S., Cani P.D. (2017). Expert Consensus Document: The International Scientific Association for Probiotics and Prebiotics (ISAPP) Consensus Statement on the Definition and Scope of Prebiotics. Nat. Rev. Gastroenterol. Hepatol..

[14] de Figueiredo F.C., de Barros Ranke F.F., de Oliva-Neto P. (2020). Evaluation of Xylooligosaccharides and Fructooligosaccharides on Digestive Enzymes Hydrolysis and as a Nutrient for Different Probiotics and Salmonella Typhimurium. LWT.

[15] Mäkeläinen H., Forssten S., Saarinen M., Stowell J., Rautonen N., Ouwehand A. (2010). Xylo-Oligosaccharides Enhance the Growth of Bifidobacteria and Bifidobacterium Lactis in a Simulated Colon Model. Benef. Microbes.

[16] Sun F., Li H., Sun Z., Liu L., Zhang X., Zhao J. (2023). Effect of Arabinoxylan and Xylo-Oligosaccharide on Growth Performance and Intestinal Barrier Function in Weaned Piglets. Animals.

[17] Su J., Zhang W., Ma C., Xie P., Blachier F., Kong X. (2021). Dietary Supplementation with Xylo-Oligosaccharides Modifies the Intestinal Epithelial Morphology, Barrier Function and the Fecal Microbiota Composition and Activity in Weaned Piglets. Front. Vet. Sci..

[18] Chen Y., Xie Y., Zhong R., Liu L., Lin C., Xiao L., Chen L., Zhang H., Beckers Y., Everaert N. (2021). Effects of Xylo-Oligosaccharides on Growth and Gut Microbiota as Potential Replacements for Antibiotic in Weaning Piglets. Front. Microbiol..

[19] Baker J.T., Duarte M.E., Holanda D.M., Kim S.W. (2021). Friend or Foe? Impacts of Dietary Xylans, Xylooligosaccharides, and Xylanases on Intestinal Health and Growth Performance of Monogastric Animals. Animals.

[20] Chen Y., Xie Y., Zhong R., Han H., Liu L., Chen L., Zhang H., Beckers Y., Everaert N. (2021). Effects of Graded Levels of Xylo-Oligosaccharides on Growth Performance, Serum Parameters, Intestinal Morphology, and Intestinal Barrier Function in Weaned Piglets. J. Anim. Sci..

[21] Liu J.B., Cao S.C., Liu J., Xie Y.N., Zhang H.F. (2018). Effect of Probiotics and Xylo-Oligosaccharide Supplementation on Nutrient Digestibility, Intestinal Health and Noxious Gas Emission in Weanling Pigs. Anim. Biosci..

[22] Pang J., Zhou X., Ye H., Wu Y., Wang Z., Lu D., Wang J., Han D. (2021). The High Level of Xylooligosaccharides Improves Growth Performance in Weaned Piglets by Increasing Antioxidant Activity, Enhancing Immune Function, and Modulating Gut Microbiota. Front. Nutr..

[23] Wang X., Xiao K., Yu C., Wang L., Liang T., Zhu H., Xu X., Liu Y. (2021). Xylooligosaccharide Attenuates Lipopolysaccharide-Induced Intestinal Injury in Piglets via Suppressing Inflammation and Modulating Cecal Microbial Communities. Anim. Nutr..

[24] Liu G., Sun W., Zhang R., Shen F., Jia G., Zhao H., Chen X., Wang J. (2024). Dietary Xylo-Oligosaccharides Alleviates LPS-Induced Intestinal Injury via Endoplasmic Reticulum-Mitochondrial System Pathway in Piglets. J. Anim. Sci..

[25] Pang J., Wang S., Wang Z., Wu Y., Zhang X., Pi Y., Han D., Zhang S., Wang J. (2021). Xylo-Oligosaccharide Alleviates Salmonella Induced Inflammation by Stimulating Bifidobacterium Animalis and Inhibiting Salmonella Colonization. FASEB J..

[26] Lee A., Stanley J.S., Mellits K.H., Connerton I.F. (2025). Prebiotic Galacto-Oligosaccharide and Xylo-Oligosaccharide Feeds in Pig Production: Microbiota Manipulation, Pathogen Suppression, Gut Architecture and Immunomodulatory Effects. Appl. Microbiol..

[27] Berndtson W.E. (1991). A Simple, Rapid and Reliable Method for Selecting or Assessing the Number of Replicates for Animal Experiments. J. Anim. Sci..

[28] Rychen G., Aquilina G., Azimonti G., Bampidis V., Bastos M.D.L., Bories G., Chesson A., Cocconcelli P.S., Flachowsky G., EFSA Panel on Additives and Products or Substances used in Animal Feed (FEEDAP) (2018). Guidance on the Assessment of the Efficacy of Feed Additives. EFSA J..

[29] Caporaso J.G., Lauber C.L., Walters W.A., Berg-Lyons D., Lozupone C.A., Turnbaugh P.J., Fierer N., Knight R. (2011). Global Patterns of 16S rRNA Diversity at a Depth of Millions of Sequences per Sample. Proc. Natl. Acad. Sci. USA.

[30] Kozich J.J., Westcott S.L., Baxter N.T., Highlander S.K., Schloss P.D. (2013). Development of a Dual-Index Sequencing Strategy and Curation Pipeline for Analyzing Amplicon Sequence Data on the MiSeq Illumina Sequencing Platform. Appl. Environ. Microbiol..

[31] Schloss P.D., Westcott S.L., Ryabin T., Hall J.R., Hartmann M., Hollister E.B., Lesniewski R.A., Oakley B.B., Parks D.H., Robinson C.J. (2009). Introducing Mothur: Open-Source, Platform-Independent, Community-Supported Software for Describing and Comparing Microbial Communities. Appl. Environ. Microbiol..

[32] Pruesse E., Quast C., Knittel K., Fuchs B.M., Ludwig W., Peplies J., Glöckner F.O. (2007). SILVA: A Comprehensive Online Resource for Quality Checked and Aligned Ribosomal RNA Sequence Data Compatible with ARB. Nucleic Acids Res..

[33] Westcott S.L., Schloss P.D. (2017). OptiClust, an Improved Method for Assigning Amplicon-Based Sequence Data to Operational Taxonomic Units. mSphere.

[34] Wang Q., Garrity G.M., Tiedje J.M., Cole J.R. (2007). Naïve Bayesian Classifier for Rapid Assignment of rRNA Sequences into the New Bacterial Taxonomy. Appl. Environ. Microbiol..

[35] Cole J.R., Wang Q., Fish J.A., Chai B., McGarrell D.M., Sun Y., Brown C.T., Porras-Alfaro A., Kuske C.R., Tiedje J.M. (2014). Ribosomal Database Project: Data and Tools for High Throughput rRNA Analysis. Nucleic Acids Res..

[36] Posit team (2023). RStudio: Integrated Development Environment for R.

[37] R Core Team (2022). R: A Language and Environment for Statistical Computing. R Foundation for Statistical Computing.

[38] Shapiro S.S., Wilk M.B. (1965). An Analysis of Variance Test for Normality (Complete Samples). Biometrika.

[39] Benjamini Y., Hochberg Y. (1995). Controlling the False Discovery Rate: A Practical and Powerful Approach to Multiple Testing. J. R. Stat. Soc..

[40] Good I.J., Toulmin G.H. (1956). The Number of New Species, and the Increase in Population Coverage, When a Sample Is Increased. Biometrika.

[41] Chao A. (1984). Nonparametric Estimation of the Number of Classes in a Population. Scand. J. Stat..

[42] Magurran A.E. Measuring Biological Diversity. https://www.wiley.com/en-gb/Measuring+Biological+Diversity-p-9781118687925.

[43] Yue J.C., Clayton M.K. (2005). A Similarity Measure Based on Species Proportions. Commun. Stat.—Theory Methods.

[44] Bray J.R., Curtis J.T. (1957). An Ordination of the Upland Forest Communities of Southern Wisconsin. Ecol. Monogr..

[45] Jaccard P. (1901). Étude Comparative de La Distribution Florale Dans Une Portion Des Alpes et Du Jura. Bull. Soc. Vaud. Sci. Nat..

[46] Excoffier L., Smouse P.E., Quattro J.M. (1992). Analysis of Molecular Variance Inferred from Metric Distances among DNA Haplotypes: Application to Human Mitochondrial DNA Restriction Data. Genetics.

[47] Anderson M.J. (2001). A New Method for Non-Parametric Multivariate Analysis of Variance. Austral Ecol..

[48] Lin H., Peddada S.D. (2020). Analysis of Compositions of Microbiomes with Bias Correction. Nat. Commun..

[49] Fernandes A.D., Macklaim J.M., Linn T.G., Reid G., Gloor G.B. (2013). ANOVA-Like Differential Expression (ALDEx) Analysis for Mixed Population RNA-Seq. PLoS ONE.

[50] Yin J., Li F., Kong X., Wen C., Guo Q., Zhang L., Wang W., Duan Y., Li T., Tan Z. (2019). Dietary Xylo-Oligosaccharide Improves Intestinal Functions in Weaned Piglets. Food Funct..

[51] Crespo-Piazuelo D., Estellé J., Revilla M., Criado-Mesas L., Ramayo-Caldas Y., Óvilo C., Fernández A.I., Ballester M., Folch J.M. (2018). Characterization of Bacterial Microbiota Compositions along the Intestinal Tract in Pigs and Their Interactions and Functions. Sci. Rep..

[52] Han G.G., Lee J.-Y., Jin G.-D., Park J., Choi Y.H., Chae B.J., Kim E.B., Choi Y.-J. (2017). Evaluating the Association between Body Weight and the Intestinal Microbiota of Weaned Piglets via 16S rRNA Sequencing. Appl. Microbiol. Biotechnol..

[53] Ding X., Lan W., Liu G., Ni H., Gu J.-D. (2019). Exploring Possible Associations of the Intestine Bacterial Microbiome with the Pre-Weaned Weight Gaining Performance of Piglets in Intensive Pig Production. Sci. Rep..

[54] Zhang W., Ma C., Xie P., Zhu Q., Wang X., Yin Y., Kong X. (2019). Gut Microbiota of Newborn Piglets with Intrauterine Growth Restriction Have Lower Diversity and Different Taxonomic Abundances. J. Appl. Microbiol..

[55] Stecher B., Chaffron S., Käppeli R., Hapfelmeier S., Freedrich S., Weber T.C., Kirundi J., Suar M., McCoy K.D., von Mering C. (2010). Like Will to Like: Abundances of Closely Related Species Can Predict Susceptibility to Intestinal Colonization by Pathogenic and Commensal Bacteria. PLoS Pathog..

[56] Karabasanavar N., Sivaraman G.K., S.P. S., Nair A.S., Vijayan A., Rajan V., P.S. G. (2022). Non-Diarrhoeic Pigs as Source of Highly Virulent and Multidrug-Resistant Non-Typhoidal Salmonella. Braz. J. Microbiol..

[57] Ding H., Zhao X., Azad M.A.K., Ma C., Gao Q., He J., Kong X. (2021). Dietary Supplementation with Bacillus Subtilis and Xylo-Oligosaccharides Improves Growth Performance and Intestinal Morphology and Alters Intestinal Microbiota and Metabolites in Weaned Piglets. Food Funct..

[58] Wang X., Tsai T., Deng F., Wei X., Chai J., Knapp J., Apple J., Maxwell C.V., Lee J.A., Li Y. (2019). Longitudinal Investigation of the Swine Gut Microbiome from Birth to Market Reveals Stage and Growth Performance Associated Bacteria. Microbiome.

[59] Corcoran B.M., Stanton C., Fitzgerald G.F., Ross R.P. (2005). Survival of Probiotic Lactobacilli in Acidic Environments Is Enhanced in the Presence of Metabolizable Sugars. Appl. Environ. Microbiol..

[60] Di Ciaula A., Garruti G., Lunardi Baccetto R., Molina-Molina E., Bonfrate L., Wang D.Q.-H., Portincasa P. (2017). Bile Acid Physiology. Ann. Hepatol..

[61] Mahowald M.A., Rey F.E., Seedorf H., Turnbaugh P.J., Fulton R.S., Wollam A., Shah N., Wang C., Magrini V., Wilson R.K. (2009). Characterizing a Model Human Gut Microbiota Composed of Members of Its Two Dominant Bacterial Phyla. Proc. Natl. Acad. Sci. USA.

[62] Looft T., Allen H.K., Cantarel B.L., Levine U.Y., Bayles D.O., Alt D.P., Henrissat B., Stanton T.B. (2014). Bacteria, Phages and Pigs: The Effects of in-Feed Antibiotics on the Microbiome at Different Gut Locations. ISME J..

[63] Rubino F., Carberry C., Waters S.M., Kenny D., McCabe M.S., Creevey C.J. (2017). Divergent Functional Isoforms Drive Niche Specialisation for Nutrient Acquisition and Use in Rumen Microbiome. ISME J..

[64] Tang S., Chen Y., Deng F., Yan X., Zhong R., Meng Q., Liu L., Zhao Y., Zhang S., Chen L. (2022). Xylooligosaccharide-Mediated Gut Microbiota Enhances Gut Barrier and Modulates Gut Immunity Associated with Alterations of Biological Processes in a Pig Model. Carbohydr. Polym..

[65] Pan J., Yin J., Zhang K., Xie P., Ding H., Huang X., Blachier F., Kong X. (2019). Dietary Xylo-Oligosaccharide Supplementation Alters Gut Microbial Composition and Activity in Pigs According to Age and Dose. AMB Express.

[66] Dowarah R., Verma A.K., Agarwal N. (2017). The Use of Lactobacillus as an Alternative of Antibiotic Growth Promoters in Pigs: A Review. Anim. Nutr..

[67] Yang Y., Shen L., Gao H., Ran J., Li X., Jiang H., Li X., Cao Z., Huang Y., Zhao S. (2021). Comparison of Cecal Microbiota Composition in Hybrid Pigs from Two Separate Three-Way Crosses. Anim. Biosci..

[68] Kubasova T., Davidova-Gerzova L., Babak V., Cejkova D., Montagne L., Le-Floc’h N., Rychlik I. (2018). Effects of Host Genetics and Environmental Conditions on Fecal Microbiota Composition of Pigs. PLoS ONE.

[69] Hu P., Niu Q., Zhu Y., Shi C., Wang J., Zhu W. (2020). Effects of Early Commercial Milk Supplement on the Mucosal Morphology, Bacterial Community and Bacterial Metabolites in Jejunum of the Pre- And Post-Weaning Piglets. Anim. Biosci..

[70] Bi Y., Cox M.S., Zhang F., Suen G., Zhang N., Tu Y., Diao Q. (2019). Feeding Modes Shape the Acquisition and Structure of the Initial Gut Microbiota in Newborn Lambs. Environ. Microbiol..

[71] Jalanka J., Cheng J., Hiippala K., Ritari J., Salojärvi J., Ruuska T., Kalliomäki M., Satokari R. (2020). Colonic Mucosal Microbiota and Association of Bacterial Taxa with the Expression of Host Antimicrobial Peptides in Pediatric Ulcerative Colitis. Int. J. Mol. Sci..

[72] Han H., Liu Z., Yin J., Gao J., He L., Wang C., Hou R., He X., Wang G., Li T. (2021). D-Galactose Induces Chronic Oxidative Stress and Alters Gut Microbiota in Weaned Piglets. Front. Physiol..

[73] Lee Y.-S., Kim T.-Y., Kim Y., Lee S.-H., Kim S., Kang S.W., Yang J.-Y., Baek I.-J., Sung Y.H., Park Y.-Y. (2018). Microbiota-Derived Lactate Accelerates Intestinal Stem-Cell-Mediated Epithelial Development. Cell Host Microbe.

[74] Gao H., Sun F., Lin G., Guo Y., Zhao J. (2022). Molecular Actions of Different Functional Oligosaccharides on Intestinal Integrity, Immune Function and Microbial Community in Weanling Pigs. Food Funct..

[75] Luo D., Li J., Xing T., Zhang L., Gao F. (2021). Combined Effects of Xylo-oligosaccharides and Coated Sodium Butyrate on Growth Performance, Immune Function, and Intestinal Physical Barrier Function of Broilers. Anim. Sci. J..

[76] Bilski J., Mazur-Bialy A., Wojcik D., Zahradnik-Bilska J., Brzozowski B., Magierowski M., Mach T., Magierowska K., Brzozowski T. (2017). The Role of Intestinal Alkaline Phosphatase in Inflammatory Disorders of Gastrointestinal Tract. Mediat. Inflamm..

[77] Bhattacharyya S., Borthakur A., Pant N., Dudeja P.K., Tobacman J.K. (2007). Bcl10 Mediates LPS-Induced Activation of NF-κB and IL-8 in Human Intestinal Epithelial Cells. Am. J. Physiol. Gastrointest. Liver Physiol..

[78] Lin R., Sun Y., Mu P., Zheng T., Mu H., Deng F., Deng Y., Wen J. (2020). *Lactobacillus Rhamnosus* GG Supplementation Modulates the Gut Microbiota to Promote Butyrate Production, Protecting against Deoxynivalenol Exposure in Nude Mice. Biochem. Pharmacol..

[79] Gonçalves P., Catarino T., Gregório I., Martel F. (2012). Inhibition of Butyrate Uptake by the Primary Bile Salt Chenodeoxycholic Acid in Intestinal Epithelial Cells. J. Cell Biochem..

[80] Moens F., Verce M., De Vuyst L. (2017). Lactate- and Acetate-Based Cross-Feeding Interactions between Selected Strains of Lactobacilli, Bifidobacteria and Colon Bacteria in the Presence of Inulin-Type Fructans. Int. J. Food. Microbiol..

[81] Liu W., Hu D., Huo H., Zhang W., Adiliaghdam F., Morrison S., Ramirez J.M., Gul S.S., Hamarneh S.R., Hodin R.A. (2016). Intestinal Alkaline Phosphatase Regulates Tight Junction Protein Levels. J. Am. Coll. Surg..

[82] Lackeyram D., Yang C., Archbold T., Swanson K.C., Fan M.Z. (2010). Early Weaning Reduces Small Intestinal Alkaline Phosphatase Expression in Pigs. J. Nutr..

