# Supplementary material for: Prebiotic Xylo-Oligosaccharides Modulate the Gut Microbiome to Improve Innate Immunity and Gut Barrier Function and Enhance Performance in Piglets Experiencing Post-Weaning Diarrhoea
Source: Microorganisms. 2025 Jul 28;13(8):1760. doi: 10.3390/microorganisms13081760 (PMC12388265; doi:10.3390/microorganisms13081760)
Supplement: Supplementary file 1 [file microorganisms-13-01760-s001.zip › microorganisms-3733487-supplementary.pdf]

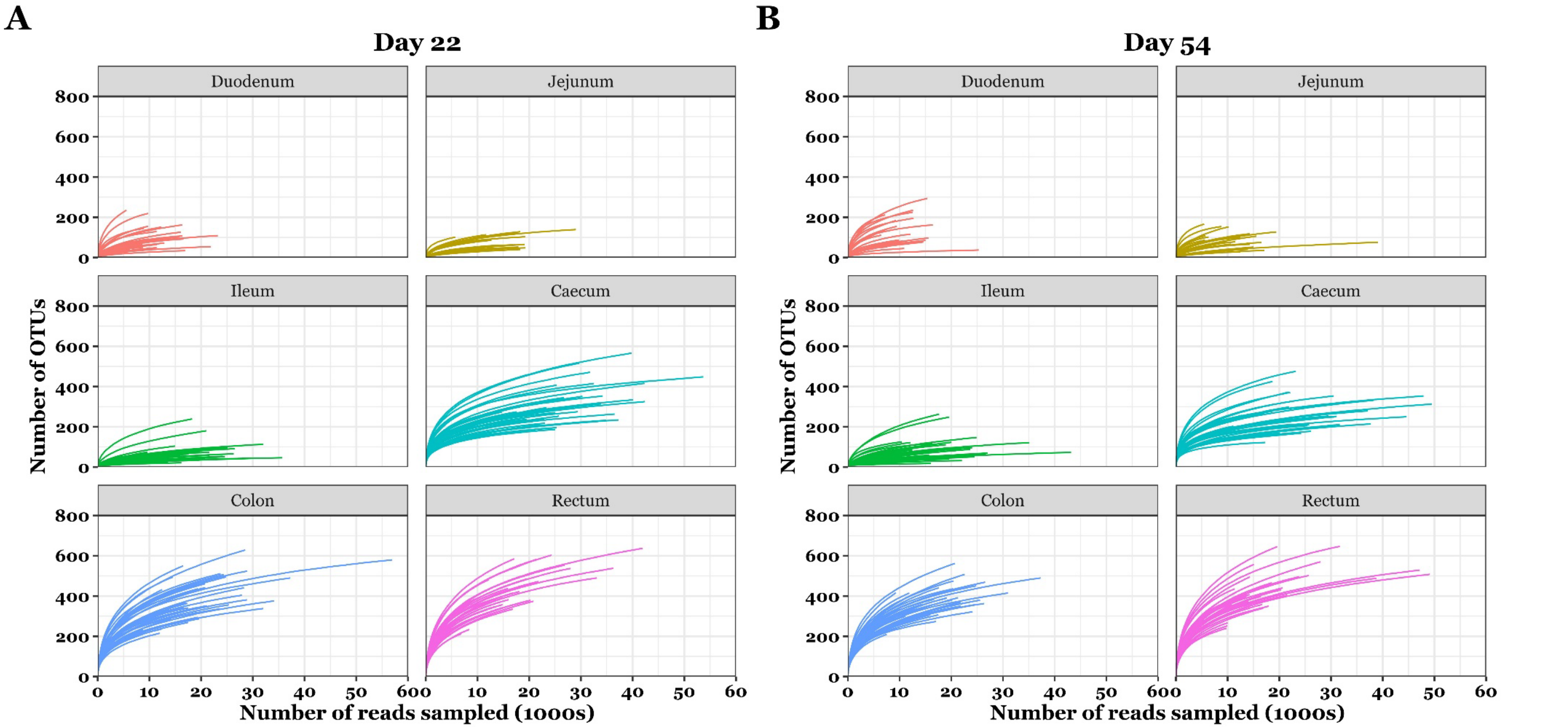

Figure S1. Sequencing effort for all communities. Sample sizes for each day and GIT location are as follows: d22 – duodenum (*n* = 28), jejunum (*n* = 29), ileum (*n* = 36), caecum *n* = 36), colon (*n* = 36), rectum (*n* = 33); d54 – duodenum (*n* = 22), jejunum (*n* = 22), ileum (*n* = 34), caecum *n* = 36), colon (*n* = 35), rectum (*n* = 35).

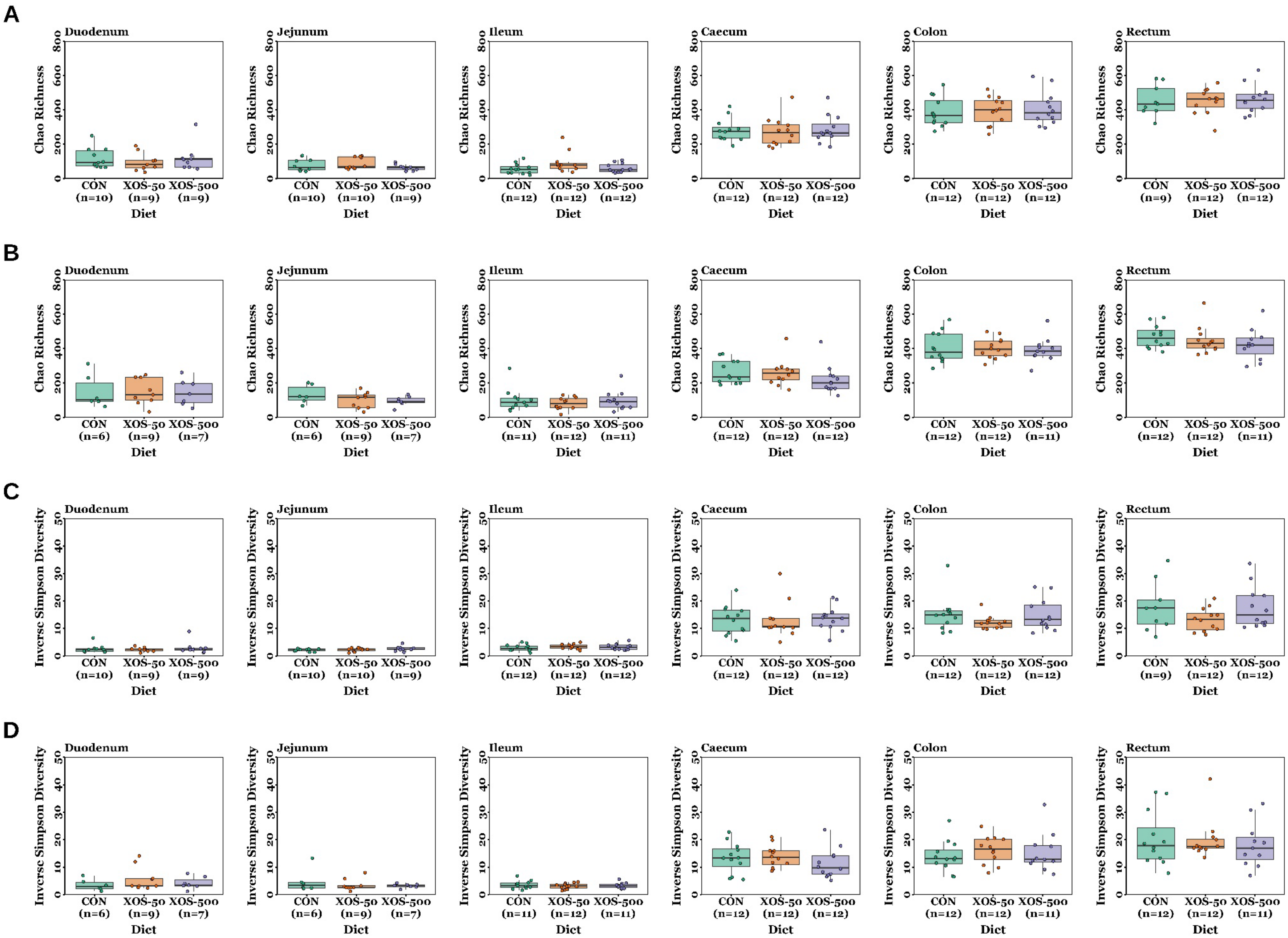

Figure S2. Boxplots showing α-diversity throughout the GIT of pigs fed control or XOS-supplemented diets. Chao richness on d22 (A) and d54 (B). Inverse Simpson diversity on d22 (C) and d54 (D).

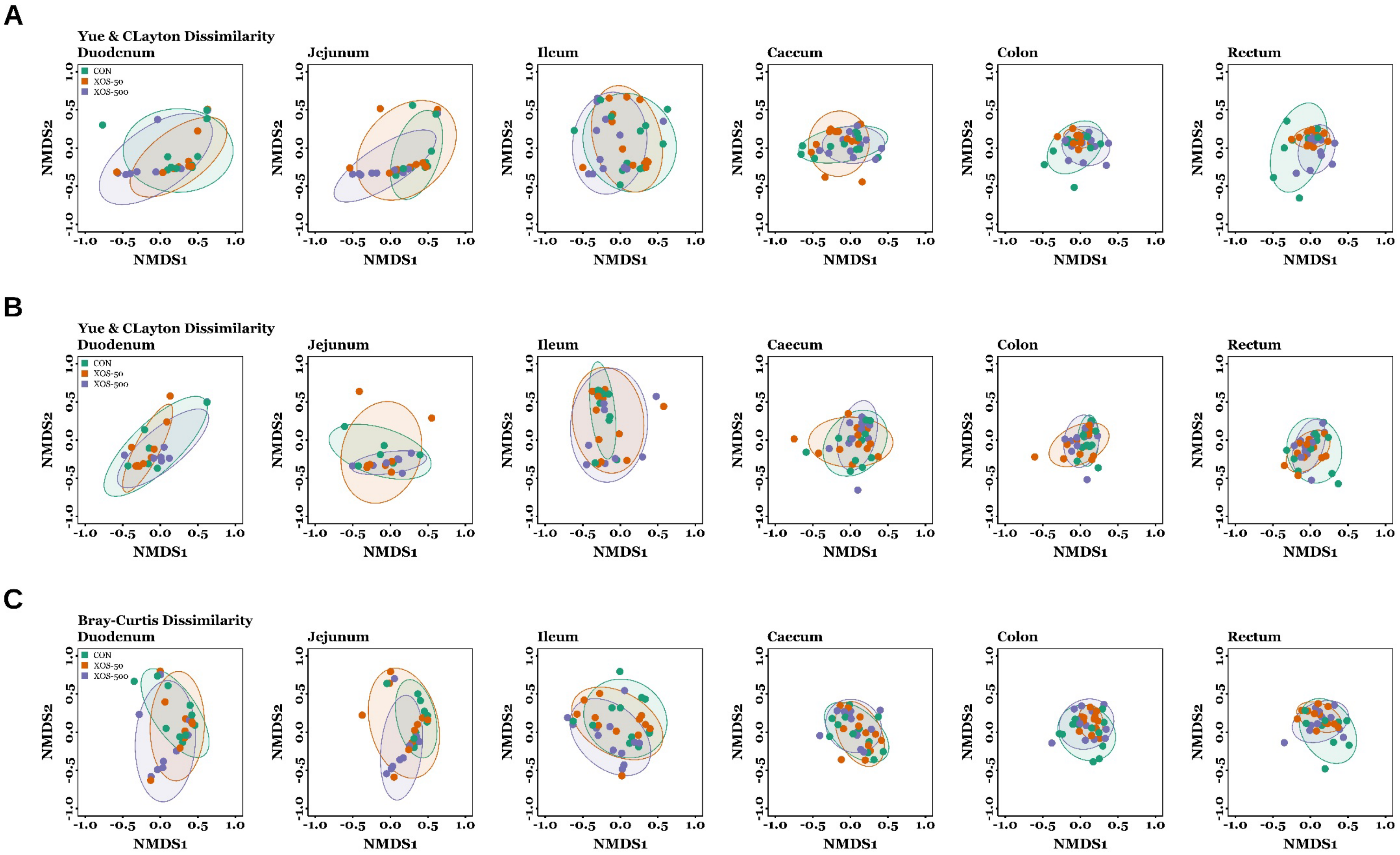
A
Yue & CLayton Dissimilarity
Duodenum
Jejunum
Ileum
Caecum
Colon
Rectum
CON
XOS-50
XOS-500
NMDS1
NMDS2
B
Yue & CLayton Dissimilarity
Duodenum
Jejunum
Ileum
Caecum
Colon
Rectum
C
Bray-Curtis Dissimilarity
Duodenum
Jejunum
Ileum
Caecum
Colon
Rectum

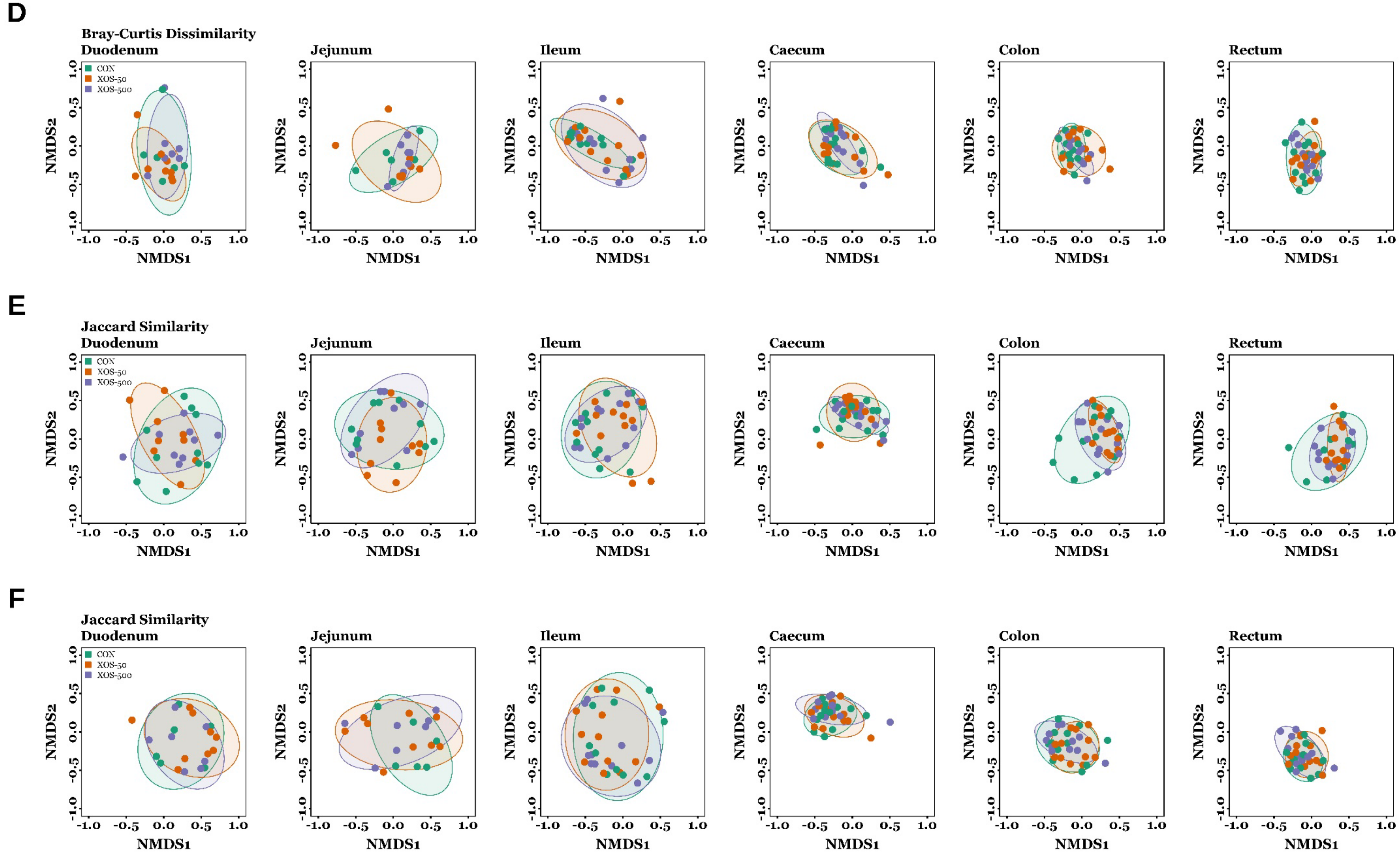

Figure S3. NMDS showing β-diversity. Yue & Clayton Dissimilarity on d22 (A) and d54 (B). Bray Curtis Dissimilarity on d22 (C) and d54 (D). Jaccard Similarity on d22 (E) and d54 (F). Sample sizes for each day, GIT location and diet group are as follows: d22 – duodenum (CON: n = 10, XOS-50: n = 9, XOS-500: n = 9), jejunum (CON: n = 10, XOS-50: n = 10, XOS-500: n = 9), ileum (CON: n = 12, XOS-50: n = 12, XOS-500: n = 12), caecum (CON: n = 12, XOS-50: n = 12, XOS-500: n = 12), colon (CON: n = 12, XOS-50: n = 12, XOS-500: n = 12), rectum (CON: n = 9, XOS-50: n = 12, XOS-500: n = 12); d54 – duodenum (CON: n = 6, XOS-50: n = 9, XOS-500: n = 7), jejunum (CON: n = 6, XOS-50: n = 9, XOS-500: n = 7), ileum (CON: n = 11, XOS-50: n = 12, XOS-500: n = 11), caecum (CON: n = 12, XOS-50: n = 12, XOS-500: n = 12), colon (CON: n = 12, XOS-50: n = 12, XOS-500: n = 11), rectum (CON: n = 12, XOS-50: n = 12, XOS-500: n = 11).

Table S1. CON and XOS feed formulations. Feed formulations for starter (d1-d7), linker (d8-d22) and grower (d23-d54) phases.

Feed component inclusion (%)	Starter phase			Linker phase			Grower phase		
	CON	XOS-50	XOS-500	CON	XOS-50	XOS-500	CON	XOS-50	XOS-500
MIC BARLEY BULK	10	10	10	15	15	15	0	0	0
Barley 62 kg/hl	0	0	0	0	0	0	15	15	15
WHEAT RAW WHOLE MEAL	20.44	20.44	20.39	37.91	37.91	37.86	47.87	47.86	47.82
MIC WHEAT MEAL BULK	10	10	10	5	5	5	0	0	0
WHEATFEED	0	0	0	0	0	0	1.03	1.03	1.03
MIC OATS	10	10	10	0	0	0	0	0	0
FISHMEAL BULK	7.25	7.25	7.25	5.77	5.77	5.77	0	0	0
SOYA HYPRO BULK	19	19	19	24	24	24	27.10	27.10	27.10
PD Pig Weaner 0.5 % 2020 (PN14934)[1]	0.5	0.5	0.5	0.5	0.5	0.5	0	0	0
DRIED SKIM MILK BULK	4	4	4	0	0	0	0	0	0
WHEY POWDER BULK	11.41	11.41	11.41	7.25	7.25	7.25	3.62	3.62	3.62
L-LYSINE HCL	0.312	0.312	0.312	0.236	0.236	0.236	0.483	0.483	0.483
L-METHIONINE	0.19	0.19	0.19	0.124	0.124	0.124	0.189	0.189	0.189
L-THREONINE	0.19	0.19	0.19	0.121	0.121	0.121	0.215	0.215	0.215
L-TRYPTOPHAN	0.029	0.029	0.029	0	0	0	0.009	0.009	0.009
L-VALINE	0.066	0.066	0.066	0	0	0	0.1	0.1	0.1
**XOS**	**0**	**0.005**	**0.05**	**0**	**0.005**	**0.05**	**0**	**0.005**	**0.05**
VITAMIN E	0.02	0.02	0.02	0.01	0.01	0.01	0.03	0.03	0.03
SUCRAM	0.01	0.01	0.01	0.01	0.01	0.01	0.01	0.01	0.01
DCP	0.84	0.84	0.84	0.97	0.97	0.97	1.95	1.95	1.95
SOYA OIL	5.74	5.74	5.74	2.94	2.94	2.94	1.68	1.68	1.68
SALT-PDV	0	0	0	0.16	0.16	0.16	0.41	0.41	0.41
ABN Pig Breeder Plain 3 (2.5)[2]	0	0	0	0	0	0	0.25	0.25	0.25
Copper sulphate	0	0	0	0	0	0	0.03	0.03	0.03
B-Traxim 2C Fe-220 (Pancosma M60-5000)	0	0	0	0	0	0	0.03	0.03	0.03

[1]Premix provides per kg of feed: 13750 IU Vitamin A; 2100 IU Vitamin D3; 150 mg Vitamin E; 6.0 mg Vitamin K3 – Menadione; 1.6 mg Vitamin B1; 6.0 mg Riboflavin; 2.3 mg Vitamin B6; 0.028 mg Vitamin B12; 25.0 mg Niacin; 13.3 mg Pantothenic acid; 1.0 mg Folic acid; 0.150 mg Biotin; 1.0 mg Iodine (calcium iodate, anhydrous); 0.25 mg Selenium (Sodium selenite); 150 mg Iron (Iron (II) sulphate monohydrate); 140 mg Copper (Copper (II) sulphate pentahydrate); 110 mg Zinc (Zinc sulphate monohydrate); 40 mg Manganese (Manganous sulphate monohydrate).

[2]Premix provides per kg of feed: 10500 IU Vitamin A; 2250 IU Vitamin D3; 50.0 mg Vitamin E; 4.0 mg Vitamin K; 0.874 mg Vitamin K3 – Menadione; 1.5 mg Vitamin B1; 4.0 mg Riboflavin; 3.5 mg Vitamin B6; 0.015 mg Vitamin B12; 20.0 mg Niacin; 12.0 mg Calcium pantothenate; 11.035 mg Pantothenic acid; 2.0 mg Folic acid; 0.2 mg Biotin; 1.0 mg Iodine (calcium iodate, anhydrous); 0.25 mg Selenium (Sodium selenite); 80.0 mg Iron (Iron (II) sulphate monohydrate); 15.0 mg Copper (Copper (II) sulphate pentahydrate); 100 mg Zinc (Zinc sulphate monohydrate); 50.0 mg Manganese (Manganous sulphate monohydrate); 10.0 mg Citric acid.

Table S2. Nutritional composition of pre-weaning creep feed offered to piglets from 15da to 28da.

Constituent	Amount/kg
Crude protein (g)	240
Crude fibre (g)	26
Crude oil and fats (g)	130
Crude ash (g)	70
Lysine (g)	16
Methionine (g)	3
Ca (g)	6
Na (g)	3.5
P (g)	6
Vitamin A (iu)	12,500
Vitamin D3 (iu)	2,000
Vitamin E (iu)	95

Table S3. Primer sequences for invA and HSP60 genes.

Target gene	Primer sequences (5′ to 3′)	Product size (bp)
invA	F: GTGCTGCTTTCTCTACTTAAC	186
	R: GAGGATTCTGTCAATGTAGAAC	
HSP60	F: CGACAAGATGGAAGCTGA	174
	R: CCACGGATCTTGTTCAAAA	
